# Supplementary material for: Proteome and Metabolome Alterations in Radish (Raphanus sativus L.) Seedlings Induced by Inoculation with Agrobacterium tumefaciens
Source: Biomolecules. 2025 Feb 14;15(2):290. doi: 10.3390/biom15020290 (PMC11852571; doi:10.3390/biom15020290)
Supplement: Supplementary file 1 [file biomolecules-15-00290-s001.zip › Supplementary_Information_1.pdf]

Proteome and Metabolome Alterations in Radish (*Raphanus sativus* L.) Seedlings Induced by Inoculation with *Agrobacterium tumefaciens*

Nadezhda Frolova, Daria Gorbach, Christian Ihling, Tatiana Bilova, Anastasia Orlova, Elena Lukasheva, Ksenia Fedoseeva, Irina Dodueva, Lyudmila A. Lutova and Andrej Frolov

Directory

Table S1-1 The settings for data acquisition and processing used in LIT-Orbitrap-MS and -MS/MS experiments...6

Table S1-2 Gas chromatographic (GC) separation conditions and electron ionization-quadrupole-mass spectrometry (EI-Q-MS) settings for analysis of *R.sativus* primary polar thermally stabile metabolites……………………………………………………………………8

Table S1-3 IP-RP-HPLC-QqQ-MS/MS instrumental settings employed for analysis of primary thermally labile metabolite of R. sativus callus...9

Table S1-4 Parameters of the target method developed using standards for the analysis of radish primary thermally labile metabolites by ion-pair reversed-phase ultra-high performance liquid chromatography-mass spectrometry (RP-IP-HPLC-QqQ-MS)………………………..15

Table S1-5 Protein recoveries and total UV densities calculated for individual samples separated by SDS-PAGE…………………………………………………………………...19

Table S1-6 The protein sequences used to design a database for targeted search of tumor- and meristem-related peptides and proteins……………………………………………………..20

Table S1-7 Thermally stable primary metabolites annotated by spectral similarity searches and/or co-elution with authentic standards in methanol extracts of radish callus by GC-MS after derivatisation of lyophilised extracts with methoxyamine hydrochloride (MOA) and N-methyl-N-(trimethylsilyl)trifluoroacetamide (MSTFA)…………………………………...…22

Figure S1-1 The numbers of tryptic peptides and possible individual proteins identified in radish (*R. sativus*) plants on the 10th (10d) and 22nd (22d) days after inoculation (d.a.i.) with cultural medium (MI) and *Agrobacterium tumefaciens* culture (AI)……………………….....29

Figure S1-2 Functional annotation of proteins, differentially expressed in *R. sativus* plants on the 22nd day after inoculation (d.a.i.) with cultural medium (mock treatment, left) and suspension of Agrobacterium tumefaciens (right) in comparison to the 10th d.a.i…………...30

Figure S1-3 Prediction of intracellular localization for the differentially expressed proteins, annotated in the tryptic digests, obtained from *R. sativus* plants on the 22th day to the corresponding 10-day treatments……………………………………………………..…31

Figure S1-4 Tandem mass spectra of unic peptides representing the proteins listed in Table 1……………………………………………………………………………………33

Figure S1-4.1 Tandem mass spectrum of the m/z 595.79 corresponding to the [M+3H]3+ ion of the peptide MGNYYSRRK representing NP_680162.1 CLAVATA3/ESR-RELATED 22 (identified with 1 PSMs, XCorr = 0.03, PEP = 0.214)……………………………….…33

Figure S1-4.2 Tandem mass spectrum of the m/z 636.59 corresponding to the [M+3H]3+ ion of the peptide SIGVQFKHTLEDQEMLNKNRR representing NP_001077799.1 CLAVATA3/ESR-RELATED 45 (identified with 4 PSMs, XCorr = 0.31, PEP = 0.153)…..34

Figure S1-4.3 Tandem mass spectrum of the m/z 464.24 corresponding to the [M+3H]3+ ion of the peptide YFGAGKFPPVDSFVGKGISESK representing NP_566783.1 CLAVATA3/ESR-RELATED 27 (identified with 4 PSMs, XCorr = 0.4, PEP = 0.0911)…34

Figure S1-4.4 Tandem mass spectrum of the *m/z* 418.55 corresponding to the $[M+3H]^{3+}$ ion of the peptide SHHQLEFLSR representing NP_190576.1 CYCLIN D3;3 (identified with 1 PSMs, XCorr = 1.25, PEP = 0.0891)…………………………………………………....35

Figure S1-4.5.1 Tandem mass spectrum of the m/z 614.82 corresponding to the [M+3H]3+ ion of the peptide NPMRGFHLEK representing NP_191622.1 GRAS family transcription factor (identified with 3 PSMs, XCorr = 0.2, PEP = 0.135)……………………………….35

Figure S1-4.5.2 Tandem mass spectrum of the m/z 580.84 corresponding to the [M+3H]3+ ion of the peptide KELVTVSAWK representing NP_191622.1 GRAS family transcription factor (identified with 1 PSMs, XCorr = 0.1, PEP =0.17)…………………………………36

Figure S1-4.6 Tandem mass spectrum of the m/z 1071.54 corresponding to the [M+3H]3+ ion of the peptide VVVFVDSEGWTEIAGSGSFR representing NP_195389.4 GRAS family transcription factor (identified with 6 PSMs, XCorr = 0.48, PEP = 0.123)………………….36

Figure S1-4.7 Tandem mass spectrum of the *m/z* 808.39 corresponding to the $[M+3H]^{3+}$ ion of the peptide AVSMGNMDSQVLLHELGFDLSK representing NP_850159.2 CLAVATA3/ESR-RELATED 5 (identified with 8 PSMs, XCorr = 0.68, PEP = 0.098)……37

Figure S1-4.8.1 Tandem mass spectrum of the *m/z* 397.21 corresponding to the $[M+3H]^{3+}$ ion of the peptide SLYYKNHHK representing NP_001318877.1 CLAVATA3/ESR-RELATED 21 (identified with 1 PSMs, XCorr =0.06, PEP = 1.2e-1)…………………………………37

Figure S1-4.8.2 Tandem mass spectrum of the *m/z* 500.25 corresponding to the $[M+3H]^{3+}$ ion of the peptide SSSIQAGRFMTTGR representing NP_001318877.1 CLAVATA3/ESR-RELATED 21 (identified with 1 PSMs, XCorr = 0.3, PEP =3.2e-1)………………………...38

Figure S1-4.9 Tandem mass spectrum of the *m/z* 1053.53 corresponding to the $[M+3H]^{3+}$ ion of the peptide HRWTPTSTQLQILESIYDEGSGTPNRR representing NP_173493.2WUSCHEL-related homeobox 14 (identified with 11 PSMs, XCorr =0.79, PEP = 0.334)………………………………………………………………………….……...38

Figure S1-4.10 Tandem mass spectrum of the *m/z* 1070.21corresponding to the $[M+3H]^{3+}$ ion of the peptide ISTELSFYGKIESKNVFYWFQNHKAR representing NP_187735.2 WUSCHEL related homeobox 5 (identified with 1 PSMs, XCorr = 0.82, PEP = 0.287)…....39

Figure S1-4.11 Tandem mass spectrum of the *m/z* 1072.52 corresponding to the $[M+3H]^{3+}$ ion of the peptide ELDQFMTHYVLLLCSFK representing NP_197904.1 homeobox protein knotted-1-like 3 (identified with 2 PSMs, XCorr = 0.46, PEP = 0.206) ……………………..39

Figure S1-4.12 Tandem mass spectrum of the m/z 595.79 corresponding to the [M+3H]3+ ion of the peptide SLLMNNGSYEEEEQVLK representing NP_683589.1 CLAVATA3/ESR-RELATED 19 (identified with 2 PSMs, XCorr = 0.47, PEP = 2.5e-1)………………………40

Figure S1-4.13 Tandem mass spectrum of the *m/z* 521.27 corresponding to the $[M+3H]^{3+}$ ion of the peptide RGRMMIEAEEVLK representing NP_001319370.1 CLAVATA3/ESR-RELATED 1 (identified with 3 PSMs, XCorr = 0.63, PEP = 2.2e-1)………………………..40

Figure S1-4.14 Tandem mass spectrum of the *m/z* 336.45 corresponding to the $[M+3H]^{3+}$ ion of the peptide IGLQIQIQSSKK representing NP_195142.1 CYCLIN D3;1 (identified with 1 PSMs, XCorr = 0.26, PEP = 0.186)………………………………………………….……41

Figure S1-4.15 Tandem mass spectrum of the *m/z* 511.27 corresponding to the $[M+3H]^{3+}$ ion of the peptide VWFQNRR representing NP_195014.1 homeobox-leucine zipper protein ATHB-8 (identified with 7 PSMs, XCorr =0.94, PEP = 0.189)……………………...……41

Figure S1-5 Comparison of the primary metabolite profiles detectable in aq. methanolic extracts prepared from the *R. sativus* calluses, cultivated on the agrobacteria cultural medium on the 10th d.a.i. - A10d and on the 22nd d.a.i. – A22d……………………………………42

Figure S1-6 Comparison of the primary metabolite profiles detectable in aq. methanolic extracts prepared from the *R. sativus* calluses harvested on the 22nd day after inoculation with a suspension of the A. tumefaciens culture (A22d) or corresponding mock-treated controls (C22d)………………………………………………………………………………………42

Tables

Table S1-1 The settings for data acquisition and processing used in LIT-Orbitrap-MS and -MS/MS experiments

Parameter	Setting
Experiment type	Data-dependent acquisition (doubly play)
**MS conditions (survey scan)**	
Ionization mode	Positive
Resolution	60.000
Ion spray voltage (IS)	1900.0 V
Capillary voltage	11.0
Capillary temperature	200 °C
Mass to charge ratio (*m/z*) range	300 – 1500
**MS/MS conditions (dependent scan)**	
Fragmentation	Collision activated dissociation
Isolation width	2.3 Da
Charge state rejected	1+
Normalized collision energy	35
Activation frequency	0.25
Activation time	30 ms
Parent mass width	± 0.5 Da
Reject mass width	± 10 ppm
Dynamic exclusion	enabled
Dynamic exclusion repeat count	1

Dynamic exclusion repeat duration	30 s
Dynamic exclusion duration	90 s
Dynamic exclusion mass width	± 10 ppm
**Database search settings**	
Analysis program	Proteome Discoverer
Search engine	Sequest
Protease	Trypsin
Missed cleavage sites	3
Modification	Mass increment (Da) / amino acids
Carbamidomethyl (cam)	+57.0215 / C
Oxidation (ox)	+15.9949 / M

Table S1-2 Gas chromatographic (GC) separation conditions and electron ionization-quadrupole-mass spectrometry (EI-Q-MS) settings for analysis of *R. sativus* primary polar thermally stabile metabolites.

Parameters	Setting<br>GC settings
Separation column	Phenomenex ZB-5MS (30 m × 0.25 mm ID, 0.25 μm film thickness)
Carrier gas / carrier gas flow rate	Helium / 1 mL/min
Injector operation mode	Splitless mode (90 s splitless time)
Injector temperature	250°C
Temperature program	1 min at 40°C<br>ramp 15°C/min to 70°C<br>1 min at 70°C<br>ramp 6°C/min to 320°C<br>12 min at 320°C
**Parameters**	**MS settings**
Ionization mode	Electron ionization (EI)
Electron energy	70 eV
Operation mode	scanning at 0.34 sec $scan^{-1}$
m/z range	50 - 700

The analysis was accomplished with Shimadzu GC2010 gas chromatograph coupled online to a quadrupole mass selective detector Shimadzu GCMS QP2010 with CTC GC PAL Liquid Injector (Shimadzu Scientific Instruments, Australia)

Table S1-3. IP-RP-HPLC-QqQ-MS/MS instrumental settings employed for analysis of primary thermally labile metabolite of *R. sativus* callus

Chromatography

ACQUITY Sample Manager (SM)	
Injection mode	PartialLoop
Injection volume	5 µL
Weak wash solvent	0.3 mmol/L aq. ammonium formate, pH 3.5 (adjusted using formic acid)
Weak wash volume	800 µL
Strong wash solvent	Acetonitrile
Strong wash volume	400 µL
Target sample temperature	4.0 C
Needle overfill flush	Automatic
**Column conditions**	
Separation column	EC 150/2 NUCLEOSHELL RP 18<br>(150 x 2 mm, particle size 2.7 µm)
Target column temperature	40.0 C
**ACQUITY Binary Solvent Manager (BSM)**	
Eluent A	10 mmol/L tributylamine (TBA) in water, pH 6.2 (adjusted using acetic
Eluent B	Acetonitrile
Seal wash duration	5 min
Flow rate	0.4 mL/min
Elution program	2% eluent B isocratic - 2 min<br>gradient to 36% eluent B – 16 min<br>gradient to 95% eluent B – 3 min<br>95% eluent B isocratic – 1.5 min<br>gradient to 2% eluent B – 0.01 min<br>2% eluent B isocratic – 3.5 min (re-equilibration)

Mass spectrometry	
**General**	
Mass analyzer type	triple quadrupole-linear ion trap (QqLIT, QTRAP, operated in QqQ mode)
Ion source	TurboIonSpray®
Experiment type	multiple reaction monitoring (MRM)
Operatinon mode	negative
Cycle time (s)	1.2
Pause between ranges (ms)	5.007
Settling time (s)	0
Duration	26 min
**Ion source settings**	
Nebulizer gas (psig)	60
Drying gas (psig)	70
Curtain gas (psig)	40
Ion spray voltage (kV)	-4.5
Ion source temperature (°C)	450
**MS/MS settings**	
Fragmentation mode	CAD
MS/MS experiment type	MRM
Collision gas	nitrogen
Collision gas pressure	3 psig (medium)
Entrance potential (V)	-10.0
Scheduled MRM	enabled
Scheduled MRM type	basic
MRM detection window (s)	500
Target scan time (s)	1
Dwell time	adjusted by scheduled MRM algorithm
Q1 resolution	unit
Q3 resolution	unit

Declustering potential (DP, V)	compound-specific (listed below)
Collision potential (CE, V)	compound-specific (listed below)
Exit potential (CXP, V)	compound-specific (listed below)

Analyte-specific settings

Analyte-specific combinations of Q1 and Q3 *m/z* ranges (transitions)

#	Analyte	t_R[a] (min)	Q1[b] (*m/z*)	Q3[c] (*m/z*)	DP (V)	CE (V)	CXP (V)
**1**	2-deoxy-*D*-ribose 5-phosphate	N/A	212.9	97.1	-40	-20	-19
**2**	3-[(carboxylatovinyl)oxy]benzoate	N/A	207.1	179.0	-240	-38	-13
**3**	5-amino-4-imidazolecarboxamide ribotide	N/A	337.1	78.9	-85	-50	-5
**4**	5-formamido-1-(5-phospho-D-ribosyl)imidazole-4-carboxamide	N/A	365.0	78.9	-40	-35	-10
**5**	5-formyl-tetrahydrofolate	N/A	472.2	315.1	-40	-35	-10
**6**	5-methyl-tetrahydrofolate	N/A	458.2	329.1	-40	-35	-10
**7**	5′-phosphoribosyl-*N*-formylglycinamide	N/A	313.0	78.9	-40	-35	-10
**8**	5'-phosphoribosyl-5-aminoimidazole	N/A	294.0	78.9	-40	-43	-10
**9**	allantoic acid	N/A	175.0	132.0	-35	-32	-12
**10**	beta-nicotinamide mononucleotide	N/A	334.0	78.9	-25	-16	-13
**11**	carboxyaminoimidazole ribotide	N/A	338.0	78.9	-40	-35	-10
**12**	chorismate	N/A	225.0	179.0	-35	-25	-10
**13**	cytidine-5'-diphosphate choline	N/A	487.0	428.0	-10	-20	-23
**14**	glycineamide ribonucleotide	N/A	285.0	78.9	-40	-35	-10
**15**	nicotinamide	N/A	121.0	76.9	-40	-16	-9
**16**	nicotinamide mononucleotide	N/A	333.0	78.9	-50	-30	-13
**17**	nicotinamide riboside	N/A	253.1	121.0	-40	-35	-10
**18**	phosphoribosylamine	N/A	227.0	78.9	-40	-35	-10
**19**	riboflavin-5′-phosphate	N/A	455.1	97.0	-35	-25	-10
**20**	succinylaminoimidazole-carboxamide ribotide	N/A	453.1	78.9	-40	-35	-10
**21**	tetrahydrofolate	N/A	444.2	176.1	-40	-35	-10
**22**	histidine	0.5	154.1	93.0	-40	-24	-3
**23**	arginine	0.6	173.1	131.0	-50	-18	-7
**24**	glutamine	0.6	145.1	108.9	-30	-18	-5
**25**	ornithine	0.6	131.1	82.9	-60	-20	-5
**26**	proline	0.6	114.1	86.0	-55	-18	-3
**27**	4-aminobutanoic acid	0.7	102.1	84.0	-35	-14	-7
**28**	alanine	0.7	88.0	41.9	-20	-20	-13
**29**	allantoin	0.7	157.0	97.2	-60	-16	-1
**30**	asparagine	0.7	131.1	87.1	-75	-16	-11
**31**	citrulline	0.7	174.1	131.0	-35	-18	-7
**32**	creatine	0.7	130.1	88.1	-25	-14	-5
**33**	cysteine	0.7	120.0	79.8	-25	-32	-2
**34**	lysine	0.7	145.1	99.0	-65	-14	-5
**35**	cystine	0.8	239.3	120.0	-40	-32	-1
**36**	dehydroascorbic acid	0.8	173.0	127.0	-15	-18	-17
**37**	glycine	0.8	74.0	74.0	-36	-13	-3
**38**	methionine	0.8	148.0	47.0	-45	-24	-5
**39**	hexoses	0.8	179.1	89.0	-50	-12	-13
**40**	S-adenosyl-*L*-homocysteine	0.8	383.1	133.9	-80	-36	-7

**41**	serine	0.8	104.0	74.0	-20	-16	-3
**42**	sucrose	0.8	341.1	89.0	-240	-38	-13
**43**	threonine	0.8	118.1	73.9	-25	-18	-3
**44**	uridine	0.8	243.1	109.9	-65	-22	-5
**45**	valine	0.8	233.3	116.0	-25	-10	-5
**46**	valine	0.8	116.1	7.0	-25	-20	-5
**47**	cytidine	1.0	242.1	108.9	-70	-18	-5
**48**	leucine + isoleucine	1.0	261.3	130.2	-30	-10	-1
**49**	leucine + isoleucine	1.0	130.1	87.1	-25	-25	-10
**50**	tyrosine	1.0	180.1	118.9	-60	-24	-5
**51**	guanosine	1.4	282.1	149.9	-80	-26	-7
**52**	2'-deoxyguanosine	1.5	266.1	150.0	-115	-24	-3
**53**	adenosine	1.5	266.1	133.9	-70	-12	-1
**54**	phenylalanine	1.5	164.1	103.0	-55	-24	-5
**55**	aspartic acid	1.9	132.0	88.0	-40	-18	-13
**56**	glucopyranonic acid	1.9	193.0	113.0	-20	-16	-5
**57**	galactopyranuronic acid	2.0	193.0	113.0	-20	-16	-5
**58**	glyoxilic acid	2.0	73.0	45.0	-25	-25	-10
**59**	*D*-galactonic acid/ *D*-gluconic acid	2.1	195.1	129.0	-50	-18	-9
**60**	glucosamine 6-phosphate	2.1	258.0	97.0	-45	-24	-5
**61**	glutamic acid	2.1	146.0	102.0	-80	-18	-9
**62**	ribonic acid	2.3	165.0	75.0	-45	-20	-35
**63**	glucosamine 1-phosphate	2.4	258.0	78.9	-55	-42	-1
**64**	2’-deoxyadenosine	2.5	250.1	134.0	-115	-26	-9
**65**	glucolate	2.5	75.0	47.0	-30	-14	-13
**66**	shikimic acid	2.5	173.0	92.9	-15	-20	-5
**67**	3-dehydroxyshikimic acid	2.7	171.0	127.0	-25	-16	-15
**68**	quinic acid	2.7	191.1	85.0	-50	-28	-13
**69**	uric acid	2.7	167.0	124.0	-45	-20	-7
**70**	ascorbic acid	2.8	175.0	115.0	-25	-25	-5
**71**	carbamoyl-alanine	2.8	131.0	87.9	-10	-14	-13
**72**	chloride	2.8	35.0	35.0	-50	-10	-10
**73**	dihydroorotic acid	3.3	157.0	112.7	-40	-10	-5
**74**	tryptophan	3.4	203.1	116.2	-50	-22	-7
**75**	lactic acid	3.8	89.0	42.9	-15	-12	-5
**76**	glutathione	4.0	306.1	143.0	-5	-26	-7
**77**	phosphate	4.0	96.9	78.9	-40	-18	-15
**78**	cyclic guanosine monophosphate	4.5	344.0	150.0	-70	-34	-11
**79**	orotic acid	4.8	155.0	110.7	-25	-12	-5
**80**	pyruvic acid	4.9	87.0	43.0	-30	-12	-1
**81**	nicotinamide adenine dinucleotide	5.0	662.1	540.1	-45	-22	-15
**82**	glucose 6-phosphate	5.4	259.1	97.0	-65	-18	-13
**83**	glyceraldehyde 3-phosphate	5.4	169.0	97.0	-30	-12	-5
**84**	fructose 6-phosphate	5.5	259.0	96.9	-30	-20	-11
**85**	2-keto-3-deoxy-6-phosphogluconate	5.8	257.0	97.0	-30	-20	-9
**86**	erythrose 4-phosphate	5.9	199.0	96.8	-40	-12	-5
**87**	adenosine 2',3'-cyclic mono-phosphate	6.2	328.0	134.0	-125	-36	-5
**88**	ribulose-5-phosphate	6.3	229.0	96.8	-35	-20	-5
**89**	glucose-1-phosphate	6.4	259.0	240.8	-30	-16	-15
**90**	ribulose-5-phosphate/xylulose-5-phosphate	6.5	229.0	96.8	-45	-18	-15
**91**	mevalonic acid lactone	6.6	147.1	59.1	-45	-20	-7
**92**	sedoheptulose 7-phosphate	6.6	289.0	97.0	-50	-22	-5
**93**	2-C-methylerythritol 4-phosphate	6.8	215.0	78.9	-40	-56	-9
**94**	glycerophosphoric acid	6.8	171.0	78.8	-45	-24	-1
**95**	cytidine monophosphate	7.0	322.2	79.0	-65	-68	-5

**96**	nicotinic acid	7.0	122.0	77.9	-55	-16	-13
**97**	pantothenic acid	7.0	218.1	88.1	-55	-18	-5
**98**	adenosine 3',5'-cyclic mono-phosphate	7.2	328.0	134.0	-125	-36	-5
**99**	ribose-1-phosphate	7.7	229.0	211.0	-50	-14	-3
**100**	uridine monophosphate	7.8	323.0	79.0	-65	-68	-5
**101**	guanosine 5'-monophosphate	8.1	362.1	78.9	-65	-66	-5
**102**	inosinic acid	8.3	347.0	134.8	-70	-38	-7
**103**	2'-deoxyguanosine 5'-monophosphate	8.4	346.1	78.8	-80	-42	-3
**104**	dihydroxyacetone phosphate	8.5	169.1	97.0	-35	-14	-11
**105**	thymidine-5'-phosphate	8.7	321.0	78.8	-65	-58	-3
**106**	1-deoxy-D-xylulose 5-phosphate	8.8	213.0	97.0	-50	-18	-1
**107**	adenosine monophosphate	8.8	346.1	78.8	-70	-52	-3
**108**	glutathione disulfide	8.9	611.1	306.1	-35	-34	-7
**109**	2'-deoxyadenosine 5'-monophosphate	9.1	330.1	195.0	-85	-22	-17
**110**	digalacturonic acid	9.5	369.1	175.0	-75	-18	-17
**111**	phosphocreatine	9.6	210.0	78.9	-35	-22	-1
**112**	malate	9.8	133.0	115.0	-20	-16	-5
**113**	succinic acid	9.9	117.0	73.0	-25	-16	-7
**114**	3-hydroxypyruvate	10.0	103.0	59.0	-30	-22	-7
**115**	4-diphosphocytidyl-2-C-methyl-*D*-erythritol	10.0	520.1	78.9	-120	-108	-9
**116**	ureidosuccinic acid	10.1	175.1	131.8	-25	-16	-7
**117**	sulfate	10.2	97.0	97.0	-40	-18	-15
**118**	uridine-5'-diphosphate-glucose	10.2	565.0	323.0	-125	-36	-11
**119**	uridine-diphosphate-*N*-acetylglucosamine	10.2	606.1	384.8	-175	-36	-25
**120**	fumaric acid	10.4	115.0	71.0	-5	-12	-13
**121**	adenosine diphosphoribose	10.5	558.1	346.0	-170	-34	-19
**122**	oxaloacetic acid-1	10.5	131.0	87.0	-35	-10	-17
**123**	oxaloacetic acid-2	10.5	131.0	43.0	-35	-18	-11
**124**	adenosine diphosphate glucose	10.6	588.1	345.9	-140	-32	-19
**125**	α-ketoglutaric acid	10.7	145.0	101	-10	-12	-13
**126**	pentanoates	10.7	101.1	101.1	-50	-10	-5
**127**	2C-methyl-*D*-erythritol 2,4-cyclodiphosphate	10.9	277.0	79.0	-45	-64	-37
**128**	(R)-5-phosphomevalonic acid	11.2	227.0	97.0	-30	-35	-10
**129**	1,4-dihydronicotinamide adenine dinucleotide	11.4	664.1	78.9	-100	-124	-1
**130**	folate	11.5	440.1	311.1	-40	-35	-10
**131**	2-phosphoglyceric acid	12.7	185.0	79.0	-25	-20	-35
**132**	isopentenyl pyrophosphate	12.8	245.0	78.9	-15	-44	-37
**133**	2*P*-glycolate	12.9	155.0	79.0	-15	-36	-35
**134**	xanthosine-5'-phosphate	12.9	363.0	151.1	-60	-36	-5
**135**	guanosine-5'-diphosphate	13.0	442.0	78.9	-85	-70	-3
**136**	6-phosphogluconic acid	13.1	275.0	79.0	-60	-66	-5
**137**	flavin adenine dinucleotide	13.1	784.1	79.0	-60	-130	-1
**138**	uridine-5'-diphosphate	13.1	403.0	78.8	-75	-68	-3
**139**	cytidine-5'-diphosphate	13.1	402.0	78.9	-65	-70	-5
**140**	3-phosphoglyceric acid	13.2	185.0	96.7	-30	-22	-7
**141**	(2E)-4-hydroxy-3-methylbut-2-en-1-yl diphosphate	13.3	261.0	79.0	-40	-52	-9
**142**	aconitic acid	13.3	173.0	128.7	-25	-10	-55
**143**	adenosine-5'-diphosphate	13.3	426.2	78.9	-75	-66	-3
**144**	nicotinamide adenine dinucleotide phosphate	13.4	743.1	620.0	-55	-22	-17
**145**	thymidine-5'-diphosphate	13.4	401.0	78.8	-70	-68	-3

**146**	2'-deoxyadenosine-5'-diphosphate	13.5	410.0	78.9	-60	-76	-3
**147**	trigalacturonic acid	13.8	545.1	369.0	-105	-24	-25
**148**	phosphoenolpyruvic acid	13.9	167.0	78.8	-20	-16	-9
**149**	isocitric acid	14.1	191.1	73.0	-45	-28	-31
**150**	citric acid	14.2	191.0	87.0	-35	-22	-15
**151**	dimethylallylpyrophosphat	14.2	245.0	78.9	-15	-44	-37
**152**	cytidine 5'-triphosphate	15.4	482.2	158.8	-85	-36	-9
**153**	4-diphosphocytidyl-2-C-methyl-*D*-erythritol 2-phosphate	15.5	600.0	78.9	-115	-126	-19
**154**	desoxyadenosintriphosphat	15.5	490.0	391.9	-90	-34	-25
**155**	sedoheptulose 1,7-bisphosphate	15.5	369.0	97.0	-35	-20	-27
**156**	adenosine triphosphate	15.6	506.2	158.8	-80	-38	-9
**157**	fructose-1,6-diphosphate	15.7	339.0	96.9	-35	-22	-11
**158**	ribulose-1,5-bisphosphate	15.7	309.0	97.0	-35	-20	-27
**159**	adenylosuccinic acid	15.8	462.3	133.9	-85	-62	-7
**160**	orotidine 5'-monophosphate	15.8	367.0	78.9	-50	-78	-1
**161**	mevalonate-5-diphosphate	15.9	307.0	78.9	-25	-35	-13
**162**	guanosine-5'-triphosphate	16.0	522.0	158.8	-90	-48	-9
**163**	deoxythymidine 5'-triphosphate	16.0	481.0	158.7	-80	-38	-9
**164**	dihydronicotinamide adenine dinucleotide phosphate	16.3	744.1	79.0	-40	-16	-9
**165**	ADP-ribose-2`-phosphate	16.5	638.0	426.0	-170	-34	-19
**166**	coenzyme A	17.0	766.1	407.9	-245	-50	-19
**167**	inositol triphosphate	17.4	419.0	320.8	-25	-28	-21
**168**	5-phosphoribosyl diphosphate	16.8	388.9	176.8	-55	-28	-9
**169**	S-acetyl coenzyme A	17.6	808.1	407.9	-220	-52	-27
**170**	methylmalonyl coenzyme A	17.8	866.1	408.0	-185	-58	-21
**171**	geranyl diphosphate	18.0	313.1	78.9	-65	-46	-1
**172**	β-hydroxy β-methylglutaryl-CoA	18.5	910.1	407.9	-220	-52	-27
**173**	malonyl coenzyme A	18.6	852.1	408.0	-185	-58	-21
**174**	ent-copal-8-ol diphosphate	19.0	467.2	78.8	-220	-52	-27
**175**	succinyl coenzyme A	19.0	866.1	407.6	-260	-56	-25
**176**	inositol-1,3,4,5-tetraphosphate	19.2	498.3	400.7	-100	-30	-27
**177**	β-methylcrotonyl coenzyme A	19.1	848.1	407.8	-185	-58	-21
**178**	1-diphosinositol pentakisphosphate	19.2	578.9	480.6	-25	-32	-31
**179**	geranylgeranyl pyrophosphate	19.2	449.2	78.8	-65	-68	-35
**180**	isovaleryl coenzyme A	19.2	850.2	407.9	-240	-58	-19
**181**	acetoacetyl coenzyme A	19.5	580.1	408.0	-220	-52	-27
**182**	Phytic acid	19.6	658.9	560.7	-145	-38	-31
**183**	farnesyl diphosphate	19.7	381.1	78.9	-50	-50	-5

The analysis relied Waters ACQUITY UPLC H-Class UPLC System (Waters GmbH, Eschborn, Germany) coupled online to a hybrid triple quadrupole-linear ion trap mass spectrometer (QqLIT) AB Sciex QTRAP 6500 (AB Sciex, Darmstadt, Germany).

[a] Retention time (t_R)

[b] *m/z* of quasi-molecular ions of standard substances

[c] *m/z* of fragment ion obtained by fragmentation of quasi-molecular ions of standard substances.

Table S1-4 Parameters of the target method developed using standards for the analysis of thermolabile metabolites by ion-pair reversed-phase ultra-high performance liquid chromatography-mass spectrometry (RP-IP-HPLC-QqQ-MS)

№	Metabolite	Q1 (*m/z*)[a]	Q3 (*m/z*)[b]	t_R (min)[c]
1	2-phosphoglyceric acid	185.0	79.0	12.72
2	3-dehydroshikimic acid	171.0	127.0	2.55
3	6-phosphoglyceric acid	275.0	79.0	12.96
4	α-ketoglutaric acid	145.0	101.0	10.29
5	allantoin	157.0	97.2	0.57
6	arginine	173.1	131.0	0.72
7	asparagine	131.0	87.0	0.56
8	aspartic acid	132.0	88.0	2.42
9	ureidosuccinic acid	175.0	131.8	9.40
10	citric acid	191.0	87.0	13.90
11	coenzyme A	766.0	408.0	17.20
12	dehydroascorbic acid	173.0	127.0	0.93
13	dihydroorotic acid	157.0	112.7	2.55
14	2-deoxyribose 5-phosphate	213.0	97.0	11.24
15	erythrose 4-phosphate	199.0	96.8	5.25
16	fructose 6-phosphate/glucoso-6-phosphate	259.0	96.9	5.56
17	fumaric acid	115.0	71.0	9.65
18	glucose-1-phosphate	259.0	240.8	5.88
19	gamma-aminobutiric acid	102.0	84.0	0.82
20	glyceraldehyde 3-phosphate	169.0	97.0	5.62
21	glutamic acid	146.0	102.0	2.42
22	glycerol 3-phosphate	171.0	78.8	5.70
23	glutamine	145.1	108.9	0.84
24	glycine	74.0	74.0	0.85
25	β-hydroxy β-methylglutaryl-CoA	910.0	408.0	18.30
26	delta3-isopentenyl pyrophosphate/dimethylallylpyrophosphat	245.0	78.9	14.25
27	isocitric acid	191.0	73.0	14.10
28	lactic acid	89.0	42.9	3.10
29	malate	133.0	115.0	9.65
30	2-*C*-methyl-*D*-erythritol 2,4-cyclic diphosphate	277.0	79.0	10.45
31	2-*C*-methylerythritol 4-phosphate	215.0	78.9	5.96
32	(*R*)-5-phosphomevalonic acid	227.0	97.0	11.44
33	mevalonate-5-diphosphate	307.0	79.0	6.45
34	orotic acid	155.0	110.0	4.00
35	pantothenic acid	218.1	88.1	6.53
36	phosphoenolpyruvic acid	167.0	78.8	13.40
37	proline	114.0	86.0	0.80

38	pyruvic acid	87.0	43.0	4.61
39	ribose-1-phosphate	229.0	96.8	6.46
40	ribulose-5-phosphate/xylulose-5-phosphate	229.0	96.8	6.70
41	sedoheptulose-1,7-biphosphate	369.0	97.0	15.76
42	serine	104.0	74.0	0.83
43	succinic acid	117.0	73.0	9.43
44	sucrose	341.0	89.0	0.85
45	threonine	118.1	73.9	0.86
46	valine	233.0	116.0	0.80
47	chorismic acid	207.0	179.0	12.32
48	oxaloacetic acid	131.0	87.0	10.20
49	2P-glycolate	185.0	79.0	12.80
50	adenosine 2',3'-cyclic mono-phosphate/adenosine 3',5'-cyclic mono-phosphate	328.0	134.0	6.73
51	3-Hydroxypyruvic acid	103.0	59.0	9.33
52	3-phosphoglyceric acid	185.0	96.7	12.72
53	S-acetyl coenzyme A	808.1	407.9	17.49
54	aconitic acid	173.0	128.7	13.14
55	adenosine	266.1	133.9	2.33
56	adenylosuccinic acid	462.1	133.9	15.87
57	adenosine-5'-diphosphate	426.0	78.9	13.17
58	adenosine diphosphate glucose	588.1	345.9	10.01
59	ADP-ribose-2`-phosphate	638.0	426.0	16.25
60	adenosine diphosphoribose	558.1	346.0	10.20
61	alanine	188.0	42.0	0.88
62	adenosine monophosphate	346.1	78.8	8.29
63	adenosine triphosphate	506.0	158.8	15.81
64	3-ureidopropionic acid/3-(carbamoylamino)propanoic acid	131.0	88.0	2.58
65	cytidine-5'-diphosphate	402.0	78.9	12.70
66	chloride	35.0	35.0	2.80
67	citrulline	174.1	131.0	0.83
68	creatine	130.0	88.0	0.57
69	phosphocreatine	210.0	78.9	8.40
70	*L*-dicysteine	239.0	120.0	1.00
71	cytidine	242.1	108.9	0.90
72	5'-deoxyadenosine	250.0	134.0	2.42
73	2'-deoxyadenosine-5'-diphosphate	410.0	78.9	12.93
74	desoxyadenosintriphosphat	490.0	391.9	15.70
75	2'-deoxyguanosine 5'-monophosphate	346.1	78.8	8.28
76	2'-deoxyguanosine	266.1	150.0	1.48
77	digalacturonic acid	369.1	175.0	10.75
78	thymidine-5'-diphosphate	401.0	78.8	12.27
79	deoxythymidine 5'-monophosphate	321.0	79.0	8.20

80	deoxythymidine 5'-triphosphate	481.0	158.7	14.70
81	1-deoxy-*D*-xylulose 5-phosphate	213.0	97.0	8.09
82	fructose-1,6-diphosphate	339.0	96.9	15.30
83	glucosamine 1-phosphate	258.0	78.9	2.44
84	glucosamine 6-phosphate	258.0	97.0	2.50
85	galactonic acid/gluconic acid	195.1	129.0	2.44
86	glucuronic/galacturnoic acid	193.0	113.0	2.50
87	glutamine	145.0	109.0	0.81
88	glycolate	75.0	47.0	2.53
89	glyoxilic acid	73.0	45.0	1.00
90	glutathione	306.1	143.0	3.85
91	guanosine-5'-triphosphate	522.0	158.8	15.50
92	guanosine	282.1	149.9	1.40
93	histidine	154.1	93.0	0.80
94	inositol triphosphate	419.9	320.8	17.30
95	inositol-1,3,4,5-tetraphosphate	498.3	400.7	18.80
96	2-keto-3-deoxy-6-phosphogluconate	257.0	97.0	5.30
97	lysine	145.1	99.0	0.90
98	malonyl coenzyme A	852.1	408.0	18.25
99	2*C*-methyl-*D*-erythritol 2,4-cyclodiphosphate/2-*C*-methyl-*D*-erythritol 2,4-cyclic diphosphate	277.0	79.0	10.46
100	β-methylcrotonyl coenzyme A	848.1	407.8	19.01
101	methionine	148.0	47.0	0.92
102	mevalonic acid lactone	147.0	59.0	6.92
103	methylmalonyl coenzyme A	866.1	408.0	17.50
104	nicotinic acid	122.0	77.9	6.42
105	nicotinamide adenine dinucleotide phosphate	743.1	620.0	13.00
106	dihydronicotinamide adenine dinucleotide phosphate	744.1	79.0	16.40
107	ornithine	131.1	82.9	0.9
108	orotidine 5'-monophosphate	367.0	78.9	15.6
109	phenylalanine	164.1	103.0	1.8
110	phosphate	96.9	78.9	3.8
111	5-phosphoribosyl diphosphate	388.9	176.8	17.5
112	quinic acid	191.1	85.0	2.6
113	ribonic acid	165.0	75.0	2.6
114	ribulose-1,5-bisphosphate	309.0	97.0	15.4
115	sedoheptulose 7-phosphate	289.0	97.0	5.8
116	S-adenosyl-*L*-homocysteine	383.1	133.9	0.9
117	shikimic acid	173	92.9	2.51
118	succinyl coenzyme A	866.1	407.6	18.5
119	sulphate	97.0	97.0	9.9
120	trigalacturonic acid	545.1	369.0	13.3
121	tryptophan	203.1	116.2	2.7

122	tyrosine	180.1	118.9	1.0
123	uric acid	167.0	124.0	2.6
124	uridine-5'-diphosphate	483.0	158.8	13.0
125	uridine-5'-diphosphate-glucose	565.0	323.0	9.5
126	uridine-diphosphate-N-acetylglucosamine	606.0	385.0	9.6
127	uridine monophosphate	323.0	79.0	7.3
128	uridine	243.1	109.9	0.9
129	xanthosine-5'-phosphate	363.0	151.1	12.8
130	folate	440.1	311.1	12.6
131	glucose	179.0	89.0	0.8
132	arginosuccinat	289.0	97.0	5.8
133	5-amino-4-imidazolecarboxamide ribotide	337.1	78.9	8.1
134	3-methylbut-2-enoyl-CoA	848.1	407.8	19.0

[a] m/z of quasi-molecular ions of standard substances

[b] m/z of fragment ion obtained by fragmentation of quasi-molecular ions of standard substances.

[c] Retention time (tR)

Table S1-5 Protein recoveries and total UV densities calculated for individual samples separated by SDS-PAGE

Sample	Protein concentration (mg/mL)	Protein recovery (mg/g fresh weight)	UV densities (AU)[a]
C10-1	7.11	2.64	14802
C10-2	2.83	1.11	15677
C10-3	3.03	1.22	14377
Ex10-1	3.87	1.34	12931
Ex10-2	6.50	2.30	16925
Ex10-3	4.96	2.31	15095
Ex10-4	9.83	2.55	17320
C22-1	1.54	0.81	16724
C22-2*	2.17	1.71	24024*
C22-3	4.37	2.36	14562
C22-4	4.50	2.31	18612
C22-5	1.45	0.63	19807
C22-6	1.81	0.81	17701
C22-7	7.56	3.03	18029
Ex22-1	8.70	2.07	20375
Ex22-2	7.92	3.49	21762
Ex22-3*	13.57	6.96	35033*
Ex22-4	9.87	3.05	17653

*denotes samples excluded from further analysis based on the outlier test (…)

Table S1-6 The protein sequences used to design a database for targeted search of tumor- and meristem-related peptides and proteins

#	Name	NCBI Reference Sequence	Description
1	CLE41	NP_566754.1	CLAVATA3/ESR (CLE)-related protein 41
2	CLE42	NP_001318356.1	CLAVATA3/ESR (CLE)-related protein 42
3	CLE44	NP_567397.1	CLAVATA3/ESR (CLE)-related protein 44
4	CLE19	NP_683589.1	CLAVATA3/ESR (CLE)-related protein 19
5	CLE1	NP_001319370.1	CLAVATA3/ESR (CLE)-related protein 1
6	CLE2	NP_193586.1	CLAVATA3/ESR (CLE)-related protein 2
7	CLE3	NP_563763.1	CLAVATA3/ESR (CLE)-related protein 3
8	CLE4	NP_850157.1	CLAVATA3/ESR (CLE)-related protein 4
9	CLE5	NP_850159.1	CLAVATA3/ESR (CLE)-related protein 5
10	CLE11	NP_683413.1	CLAVATA3/ESR (CLE)-related protein 11
11	CLE12	NP_564943.1	CLAVATA3/ESR (CLE)-related protein 12
12	CLE16	NP_001318176.1	CLAVATA3/ESR (CLE)-related protein 16
13	CLE17	NP_565004.1	CLAVATA3/ESR (CLE)-related protein 17
14	CLE18	NP_683472.1	CLAVATA3/ESR (CLE)-related protein 18
15	CLE21	NP_001318877.1	CLAVATA3/ESR (CLE)-related protein 21
16	CLE22	NP_680162.1	CLAVATA3/ESR (CLE)-related protein 22
17	CLE25	NP_683600.1	CLAVATA3/ESR (CLE)-related protein 25
18	CLE26	NP_177155.1	CLAVATA3/ESR (CLE)-related protein 26
19	CLE27	NP_566783.1	CLAVATA3/ESR (CLE)-related protein 27
20	CLE45	NP_001077799.1	CLAVATA3/ESR (CLE)-related protein 45
21	CLE46	XP_020886768.1	CLAVATA3/ESR (CLE)-related protein 46
22	WOX4	NP_175145.2	WUSCHEL-related homeobox 4
23	WOX5	NP_187735.1	WUSCHEL-related homeobox 5

24	WOX9	NP_180944.2	WUSCHEL-related homeobox 9
25	WOX14	NP_173493.1	WUSCHEL-related homeobox 14
26	HAM2	NP_191622.1	Scarecrow-like protein 22
27	HAM4	NP_195389.4	Scarecrow-like protein 15
28	KNAT1	NP_192555.1	Homeobox protein knotted-1-like 1
29	KNAT2	NP_177208.2	Homeobox protein knotted-1-like 2
30	KNAT3	NP_197904.1	Homeobox protein knotted-1-like 3
31	KNAT4	NP_196667.2	Homeobox protein knotted-1-like 4
32	KNAT5	NP_194932.1	Homeobox protein knotted-1-like 5
33	ATHB8	NP_195014.1	Homeobox-leucine zipper protein ATHB-8
34	ANT	NP_195489.1	AP2-like ethylene-responsive transcription factor ANT
35	CYCD3;1	NP_195142.1	Cyclin-D3-1
36	CYCD3;2	NP_201527.1	Cyclin-D3-2
37	CYCD3;3	NP_190576.1	Cyclin-D3-3

Table S1-7 Thermostable primary metabolites annotated by spectral similarity searches and/or co-elution with authentic standards in methanol extracts of radish callus by GC-MS after derivatisation of lyophilised extracts with methoxyamine hydrochloride (MOA) and N-methyl-N-(trimethylsilyl)trifluoroacetamide (MSTFA)

#	Analyte[a]	Derivative[b]	t_R exp[c]	RI[d]	$m/z_{quant.}$[e]	Annotation[f]
1	Boric acid	3TMS	7.73	1009.8	221	NIST (769)
2	RI1045 Amine (Aminomethanol 3TMS)		8.53	1044.9	174	
3	2,3-Butanediol	2TMS	8.62	1048.7	117	NIST (845)
4	4-Hydroxypyridine	1TMS	8.75	1054.4	155	NIST (932)
5	RI1072 Unknown		9.17	1072.5	234	
6	Lactic acid	2TMS	9.34	1079.9	117	NIST (705)
7	**Glycolic acid**	2TMS	9.68	1095.1	177	ASL
8	**Valine**	1TMS	9.97	1107.2	72	ASL
9	**Alanine 1L**	2TMS	10.12	1113.6	116	ASL
10	**Alanine 2H**	2TMS	10.22	1117.6	116	ASL
11	Hydroxylamine	3TMS	10.32	1121.9	249	NIST (909)
12	RI1126 Unknown		10.41	1125.8	155	
13	**Glycine**	2TMS	10.64	1135.2	102	ASL
14	**Oxalic acid**	2TMS	10.84	1143.9	190	ASL
15	RI1162 Unknown		11.26	1161.8	132	
16	**Leucine**	1TMS	11.42	1168.4	86	ASL
17	**Isoleucine**	1TMS	11.84	1186.3	86	ASL
18	**Valine**	2TMS	12.63	1218.6	144	ASL
19	5-Nonanol	1TMS	12.88	1228.7	159	NIST (755)
20	**Urea**	2TMS	13.40	1249.8	189	ASL
21	**Benzoic acid**	1TMS	13.44	1251.3	179	ASL
22	**Serine**	2TMS	13.57	1256.7	116	ASL
23	Ethanolamine	3TMS	13.71	1262.5	174	NIST (932)
24	**Octanoic acid (C8:0)**	1TMS	13.77	1265.0	201	ASL
25	Phosphate	3TMS	13.88	1269.3	299	NIST (955)
26	**Leucine**	2TMS	13.88	1269.4	158	ASL
27	**Glycerol**	3TMS	13.93	1271.3	205	ASL
28	**Isoleucine**	2TMS	14.35	1288.2	158	ASL
29	**Threonine**	2TMS	14.38	1289.6	219	ASL
30	**Proline**	2TMS	14.43	1291.4	142	ASL
31	**Nicotinic acid**	1TMS	14.52	1295.0	180	ASL
32	**Maleic acid**	2TMS	14.61	1298.8	245	ASL
33	**Glycine**	3TMS	14.61	1298.8	174	ASL
34	**Succinic acid**	2TMS	14.85	1309.6	247	ASL
35	**Glyceric acid**	3TMS	15.18	1324.8	189	ASL
36	RI1325 Unknown		15.19	1325.2	184	
37	Uracil	2TMS	15.34	1332.0	241	NIST (780)

38	Itaconic acid	2TMS	15.46	1337.7	215	
39	**Fumaric acid**	2TMS	15.66	1346.7	245	ASL
40	Alanine	3TMS	15.76	1351.1	188	NIST (825)
41	**Serine 3TMS**	3TMS	15.83	1354.7	204	ASL
42	Nonanoic acid	1TMS	15.89	1357.2	117	NIST (647)
43	3-cyano-Alanine	2TMS	16.09	1366.6	141	NIST (621)
44	**Threonine**	3TMS	16.38	1379.7	218	ASL
45	RI1389 Amine		16.58	1389.0	174	
46	Methionine	1TMS	16.98	1407.3	104	NIST (816)
47	S-methyl-Cysteine	2TMS	17.13	1414.7	218	NIST (689)
48	**Aspartic acid**	2TMS	17.24	1419.6	160	ASL
49	**β-Alanine**	3TMS	17.26	1420.7	248	ASL
50	RI1433 Unknown		17.52	1433.0	243	
51	RI1443 Unknown		17.75	1443.7	259	
52	RI1445 Unknown		17.79	1445.4	350	
53	Ornithine-1,5-lactam	2TMS	17.86	1449.1	128	GDM (703)
54	**Decanoic acid (C10:0)**	1TMS	17.96	1453.5	117	ASL
55	**Glutamine [-H_2O]**	2TMS	18.23	1466.4	155	ASL
56	RI1472 Sugar related		18.36	1472.5	234	
57	**Malic acid**	3TMS	18.55	1481.5	233	ASL
58	RI1487 Unknown		18.67	1487.3	234	
59	Asparagine [-H_2O]	2TMS	18.83	1494.7	115	NIST (951)
60	**Pyroglutamic acid**	1TMS	18.88	1497.0	84	NIST (539), ASL
61	**Salicylic acid**	2TMS	18.94	1500.1	267	ASL
62	**meso-Erythritol**	4TMS	18.95	1500.3	217	ASL
63	Putrescine	3TMS	18.98	1502.0	174	GDM (743)
64	Methionine	2TMS	19.14	1510.1	176	ASL
65	**Pyroglutamic acid**	2TMS	19.19	1512.7	156	ASL
66	**Aspartic acid**	3TMS	19.20	1512.7	232	ASL
67	**γ-Aminobutyric acid**	3TMS	19.35	1520.8	174	ASL
68	**Glutamic acid**	2TMS	19.47	1526.9	174	ASL
69	**Erythronic acid**	4TMS	19.65	1536.1	292	ASL
70	**Phenylalanine**	1TMS	19.77	1541.8	120	ASL
71	**Cysteine**	3TMS	19.87	1547.0	218	ASL
72	L-Threonic acid	4TMS	20.00	1553.4	292	NIST (736)
73	Serine	4TMS	20.19	1563.2	243	NIST (558), ASL
74	**α-ketoglutaric acid**	1MEOX, 2TMS	20.33	1570.2	198	ASL
75	RI1572 Unknown		20.37	1572.4	291	
76	Proline [+CO_2]	2TMS	20.40	1573.8	142	GDM (681)
	RI1578 Amine		20.48	1578.0	174	
77	RI1582 C5-sugar (furanose)		20.56	1582.2	217	

78	Asparagine	2TMS	20.75	1591.8	159	NIST (772)
79	RI1593 Unknown		20.79	1593.5	161	
80	RI1596 Unknown		20.85	1596.4	217	
81	**Glutamic acid**	3TMS	21.16	1612.7	246	ASL
82	**Phenylalanine**	2TMS	21.24	1617.3	218	ASL
83	2-Imidazolidone-4-carboxylic acid	3TMS	21.33	1621.9	229	GDM (635)
84	**Tartaric acid**	4TMS	21.50	1630.9	292	ASL
85	**Phloroglucinol**	3TMS	21.63	1637.7	342	ASL
86	**Xylose 1L**	1MEOX, 4TMS	21.66	1639.5	307	ASL
87	**Xylose 2H**	1MEOX, 4TMS	21.83	1648.7	307	ASL
88	**Arabinose**	1MEOX, 4TMS	21.96	1655.7	307	ASL
89	RI1659 Phloroglucinol derivative		22.03	1659.0	342	
90	RI1664 Unknown		22.12	1664.0	261	
91	RI1667 Amino acid derivate		22.18	1667.0	218	
92	**Ribulose&Xylulose**	1MEOX, 4TMS	22.22	1669.1	263	ASL
93	**Ribose**	1MEOX, 4TMS	22.24	1670.4	307	ASL
94	RI1679 Unknown		22.41	1679.3	140	
95	3,5-Di-*tert*-butylphenol	1TMS	22.46	1682.3	277	NIST (816)
96	RI1691 C5-sugar alcohol		22.58	1688.3	307	
97	RI1695 fatty acid or alkane		22.63	1690.6	97	
98	1,6-Anhydro-β-d-glucose	3TMS	22.70	1694.9	204	NIST (749)
99	RI1702 Unknown		22.84	1702.2	227	
100	Arabitol (ISD)	5TMS	23.10	1716.9	307	NIST (945)
101	**Putrescine**	4TMS	23.28	1726.9	174	ASL
102	**Arginine [-NH_3]**	2TMS	23.47	1737.6	184	ASL
103	**Ornithine**	3TMS	23.57	1743.3	174	ASL
104	**Glycerol 3-phosphate**	4TMS	23.80	1756.1	357	ASL
105	Glucopyranose [-H_2O]	4TMS	23.89	1761.2	217	GDM (708)
106	Arabinonic acid	5TMS	24.10	1772.7	292	GDM (660)
107	Ethanolaminephosphate	4TMS	24.13	1774.8	174	GDM (690)
108	1,4-Benzenedicarboxylic acid	2TMS	24.30	1783.9	295	NIST (873)
109	Fructopyranose	5TMS	24.51	1795.6	217	GDM (696)
110	**Shikimic acid**	4TMS	24.66	1804.3	204	ASL
111	**Ornithine**	4TMS	24.72	1808.1	142	ASL
112	**Citric acid**	4TMS	24.78	1811.4	273	ASL
113	**Dehydroascorbic acid dimer 1L**	2MEOX	25.14	1832.3	316	ASL
114	**Dehydroascorbic acid dimer 2H**	2MEOX	25.23	1838.1	316	ASL
115	RI1841 Unknown		25.28	1840.7	188	
116	RI1845 Unknown		25.35	1844.8	285	
117	**Lysine**	3TMS	25.35	1845.2	174	ASL
118	Asparagine	4TMS	25.61	1860.3	188	

119	**Fructose 1H**	1MEOX, 5TMS	25.66	1863.3	307	ASL
120	RI1867 C6-sugar derived acid		25.73	1867.4	292	
121	**Fructose 2H**	1MEOX, 5TMS	25.83	1873.2	307	ASL
122	**Mannose 1H**	1MEOX, 5TMS	25.91	1877.5	319	ASL
123	**Galactose 1H**	1MEOX, 5TMS	25.97	1881.4	319	ASL
124	Glucopyranose	5TMS	26.01	1883.8	204	GDM (571)
125	**Glucose 1H**	1MEOX, 5TMS	26.09	1888.2	319	ASL
126	**Glucose 2L**	1MEOX, 5TMS	26.39	1906.6	319	ASL
127	**cys-Ferulic acid**	2TMS	26.52	1914.1	338	ASL
128	**Mannitol**	6TMS	26.63	1921.0	319	ASL
129	RI1925 Unknown		26.70	1925.5	275	
130	**Glucuronic acid 1H**	1MEOX, 4TMS	26.71	1926.1	333	ASL
131	**Sorbitol**	6TMS	26.74	1927.9	319	ASL
132	RI1925 C6-sugar		26.77	1929.8	204	
133	*p*-Coumaric acid	2TMS	26.88	1936.3	293	NIST (528)
134	**Ascorbic acid**	4TMS	26.95	1940.3	332	ASL
135	RI1945 C6-sugar		27.02	1945.2	249	
136	RI1945 Unknown		27.06	1947.4	204	
137	**Gallic acid**	4TMS	27.10	1949.8	281	ASL
138	RI1954 C6-sugar derived acid		27.17	1954.3	333	
139	RI1975 C6-sugar		27.51	1975.1	204	
140	RI1986 C6-sugar derived acid		27.70	1986.5	292	
141	**citric acid**	6TMS	27.76	1990.3	333	ASL
142	**Glucaric acid**	6TMS	27.92	2000.3	333	ASL
143	RI12005 C6-sugar derived acid		28.00	2005.4	333	
144	RI2009 C6-sugar		28.06	2009.0	155	
145	RI2023 C6-sugar		28.28	2023.6	204	
146	**Galactaric acid**	6TMS	28.46	2035.1	333	ASL
147	**Palmitic acid (C16:0)**	1TMS	28.56	2041.3	313	ASL
148	*cis*-Sinapinic acid	2TMS	28.74	2052.8	368	NIST (611)
149	**N-Acetyl-D-glucosamine 1L**	1MEOX, 4TMS	28.96	2067.2	319	ASL
150	**N-Acetyl-D-glucosamine 2H**	1MEOX, 4TMS	29.07	2074.0	319	ASL
151	**Myo-inositol**	6TMS	29.18	2081.4	318	ASL
152	***trans*-Ferulic acid**	2TMS	29.31	2089.3	338	ASL
153	RI2117 Unknown		29.73	2117.0	290	
154	RI2128 Unknown		29.88	2127.6	156	
155	***trans*-Caffeic acid**	3TMS	29.97	2133.5	219	ASL
156	Heptadecanoic acid (C17:0)	1TMS	30.09	2141.6	327	NIST (501)
157	1-Octadecanol	1TMS	30.26	2153.0	327	NIST (518)
158	RI2169 Unknown		30.50	2169.1	204	

159	RI2173 C6-7-sugar derivative		30.57	2173.7	361	
160	RI2182 C6-7-sugar derivative		30.69	2181.7	204	
161	RI2184 C6-7-sugar derivative		30.73	2184.2	235	
162	RI2190 C6-7-sugar derivative		30.82	2190.0	204	
163	Glycerophosphoglycerol	5TMS	30.88	2193.9	357	NIST (625)
164	**Tryptophan**	2TMS	30.99	2201.6	202	ASL
165	Linoleic acid (C18:2)	1TMS	31.06	2206.3	337	NIST (788)
166	Oleic acid (C18:1)	1TMS	31.15	2212.4	339	NIST (735)
167	*cis*-13-Octadecenoic acid (C18:1n-5)	1TMS	31.26	2220.2	339	NIST (699)
168	Stearic acid (C18:0)	1TMS	31.54	2240.1	341	NIST (958)
169	*trans*-Sinapic acid	2TMS	31.57	2242.2	368	
170	RI2250 Sugar phosphate		31.69	2250.1	315	
171	RI2271 Unknown		31.99	2270.9	290	
172	**Fructose-6-phosphate**	1MEOX, 6TMS	32.42	2301.0	315	ASL
173	**Glucose-6-phosphate 1H**	1MEOX, 6TMS	32.57	2312.1	387	ASL
174	**Glucose-6-phosphate 2L**	1MEOX, 6TMS	32.81	2329.3	387	ASL
175	RI2353 C6-7-sugar derivative		33.13	2353.1	262	
176	RI2360 C6-7-sugar derivative		33.24	2360.5	204	
177	RI2370 C6-7-sugar derived acid		33.36	2369.6	292	
178	RI2382 Sugar phosphate		33.54	2382.3	387	
179	Adipic acid, bis(2-ethylhexyl) ester		33.63	2389.1	129	NIST (831)
180	Myo-inositol phosphate	7TMS	33.84	2404.6	318	NIST (650)
181	RI2420 C6-7-sugar derivate		34.05	2420.0	361	
182	RI2433 Mono-or disaccharide		34.23	2433.5	204	
183	Arachidic acid (C20:0)	1TMS	34.30	2439.4	369	NIST (644)
184	RI2444 Fatty acid		34.37	2444.0	325	
185	RI2493 Unknown		35.01	2492.7	288	
186	RI2494 Fatty acid		35.02	2493.8	276	
187	RI2497 Mono- or disaccharide		35.07	2497.1	204	
188	RI2503 Mono- or disaccharide		35.15	2502.9	361	
189	RI2507 Mono- or disaccharide		35.20	2507.0	204	
190	RI2513 Mono- or disaccharide		35.28	2513.2	361	
191	RI2521 Mono- or disaccharide		35.38	2521.3	361	
192	RI2551 Mono- or disaccharide		35.76	2550.9	361	
193	RI2580 Mono- or		36.13	2579.7	361	

	disaccharide					
194	RI2589 Unknown		36.25	2589.5	283	
195	**Sucrose**	8TMS	36.73	2628.3	437	ASL
196	RI2667 Disaccharide		37.21	2667.3	204	
197	Cellobiose 1H	1MEOX, 8TMS	37.35	2678.9	204	GDM (734)
198	RI2693 Disaccharide		37.53	2692.9	361	
199	Cellobiose 2L	1MEOX, 8TMS	37.57	2696.3	204	GDM (734)
200	RI2702 Disaccharide		37.63	2701.7	361	
201	RI2713 Disaccharide		37.77	2713.2	204	
202	RI2718 Disaccharide		37.82	2717.6	361	
203	**Maltose 1H**	1MEOX, 8TMS	37.95	2728.5	361	ASL
204	**D-Trehalose α,α'**	8TMS	38.09	2739.5	361	ASL
205	**Maltose 2L**	1MEOX, 8TMS	38.28	2755.7	361	ASL
206	RI2764 Disaccharide		38.37	2763.6	361	
207	RI2788 Unknown		38.66	2787.6	311	
208	trans-Squalen		38.96	2813.0	69	NIST (801)
209	RI2818 Disaccharide		39.02	2818.4	361	
210	RI2959 Unknown		40.61	2958.6	340	
211	**Galactinol**	9TMS	40.83	2978.2	204	ASL
212	Galactinol derivate		41.07	3000.5	204	
213	RI3018 Disaccharide		41.27	3018.3	361	
214	Hexacosanoic acid (C26:0)	1TMS	41.44	3034.5	453	NIST (770)
215	RI3046 Disaccharide		41.57	3046.3	361	
216	RI3064 Disaccharide		41.76	3064.4	361	
217	Cholesterol	1TMS	42.64	3147.3	368	
218	RI3155 Unknown		42.72	3155.0	340	
219	RI3230 Disaccharide		43.49	3230.1	361	
220	RI3276 Disaccharide		43.96	3276.4	160	
221	β-Sitosterol	1TMS	44.61	3341.5	357	NIST (545)
222	RI3351 Unknown		44.71	3351.3	340	
223	RI3546 Unknown		46.66	3546.3	340	
224	RI3576 Unknown		46.98	3576.4	316	

[a] Analytes (metabolite features) detected by GC-MS are arranged in order of increasing retention times (t_R). Bold fond marks metabolite features identified by coelution with authentic standards. Structural annotation of other analytes relied on GS-MS data of known spectral libraries such a s NIST and Golm metabolomic database (GDM). 1 and 2 mark peaks of isomers. L and H indicate relative abundance of the isomeric peaks such as low and high, respectively. Analytes which structure could not be established with help of MS-libraries were annotated to a certain chemical class by the presence of characteristic fragment ions (*m/z* values) in the analyte' electron ionization mass-spectra as follows: *m/z* 361, 437, 451 indicate di- and oligosaccharides, *m/z* 361, 204, 433 are characteristic for galactinol, *m/z* 299, 315, 357, and 387 are characteristic for sugar phosphates, *m/z* 319 and 204 are indicative for C6-aldoses; *m/z* 318 and 319 mark cycled polyols; *m/z* 233, 292, and 319 are diagnostic for most sugar derived acids. Annotations of the analytes start by their specific retention index (RI). Analytes which could not be annotated to any chemical class are labeled with the word "*Unknown*", their annotations also is indicated by their unique RI value.

[b] Number and type of derivatization groups of identified or structurally-annotated metabolite features: TMS - trimethylsilyl group, MEOX - methyloxime group.

[c] Retention time of the analyte;

[d] Retention index of the analyte;

[e] The *m/z* value of the most characteristic ion in the analyte EI spectrum for which extracted ion chromatogram integration was performed at the given t_R;

[f] Annotation was performed according NIST or GMD library (with indication of match factor in parenthesis) and authentic standard library (ASL) with use of coeluted indicated authentic standards.

Figures

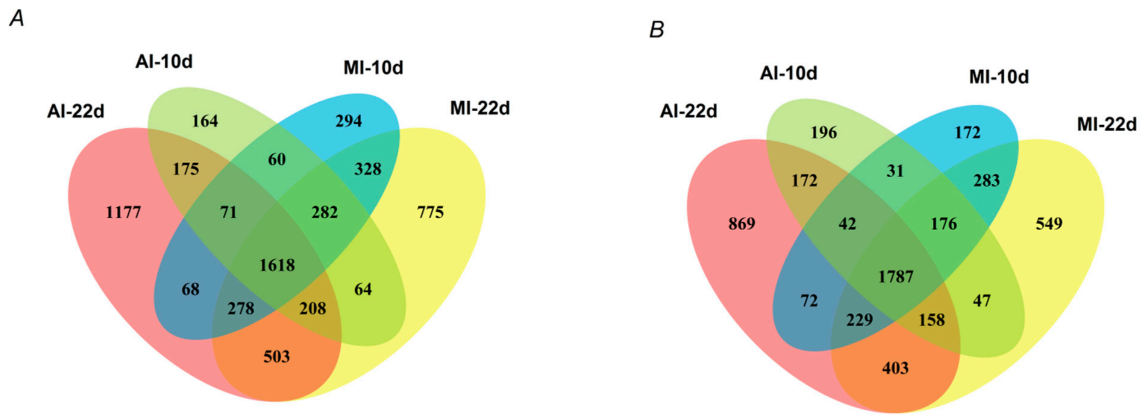

Figure S1-1 The numbers of tryptic peptides (A) and possible individual proteins (B) identified in radish (*Raphanus sativus*) plants on the 10th (10d) and 22nd (22d) days after inoculation (d.a.i.) with cultural medium (MI) and *Agrobacterium tumefaciens* culture (AI). The tryptic digests (n = 3), obtained from radish seedlings, were analyzed by nano-high performance liquid chromatography-electrospray ionization linear ion trap-orbital trap mass spectrometry (nanoHPLC-ESI-LIT-Orbitrap-MS) in DDA mode.

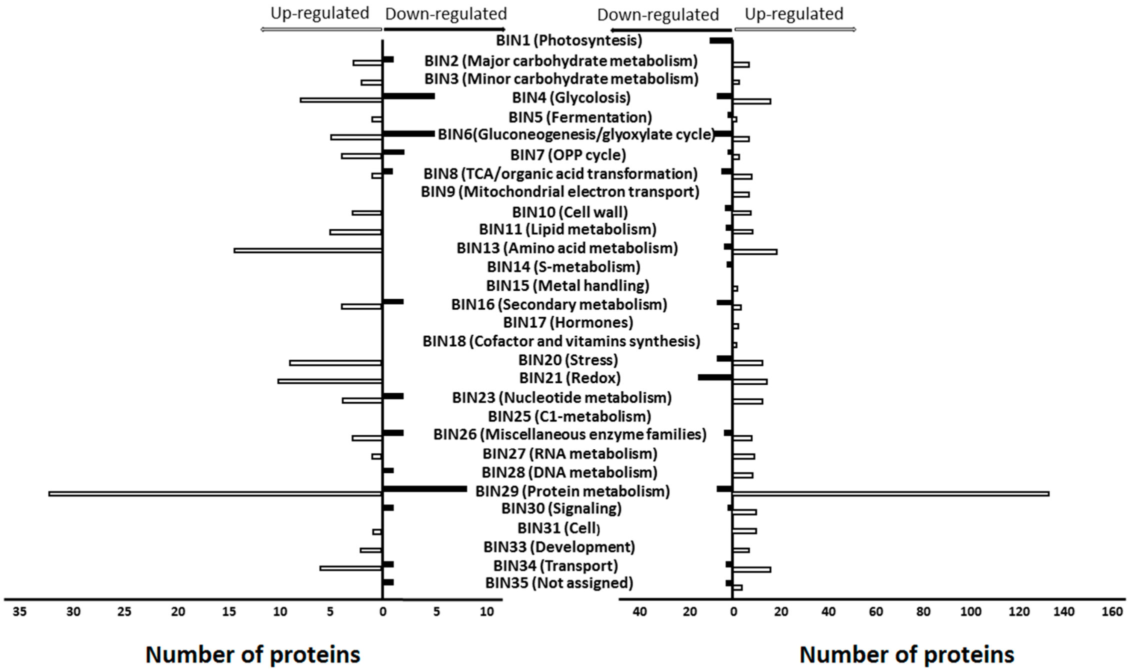

Figure S1-2 Functional annotation of proteins, differentially expressed in *Raphanus sativus*plants on the 22nd day after inoculation (d.a.i.) with cultural medium (mock treatment, left) and suspension of *Agrobacterium tumefaciens* (right) in comparison to the 10th d.a.i. White and black boxes denote the proteins, up- and down-regulated, respectively, on the 22nd d.a.i. in comparison to the 10th d.a.i. Functional annotation relied on MapMan annotation with subsequent manual curation of data based on literature and database entries.

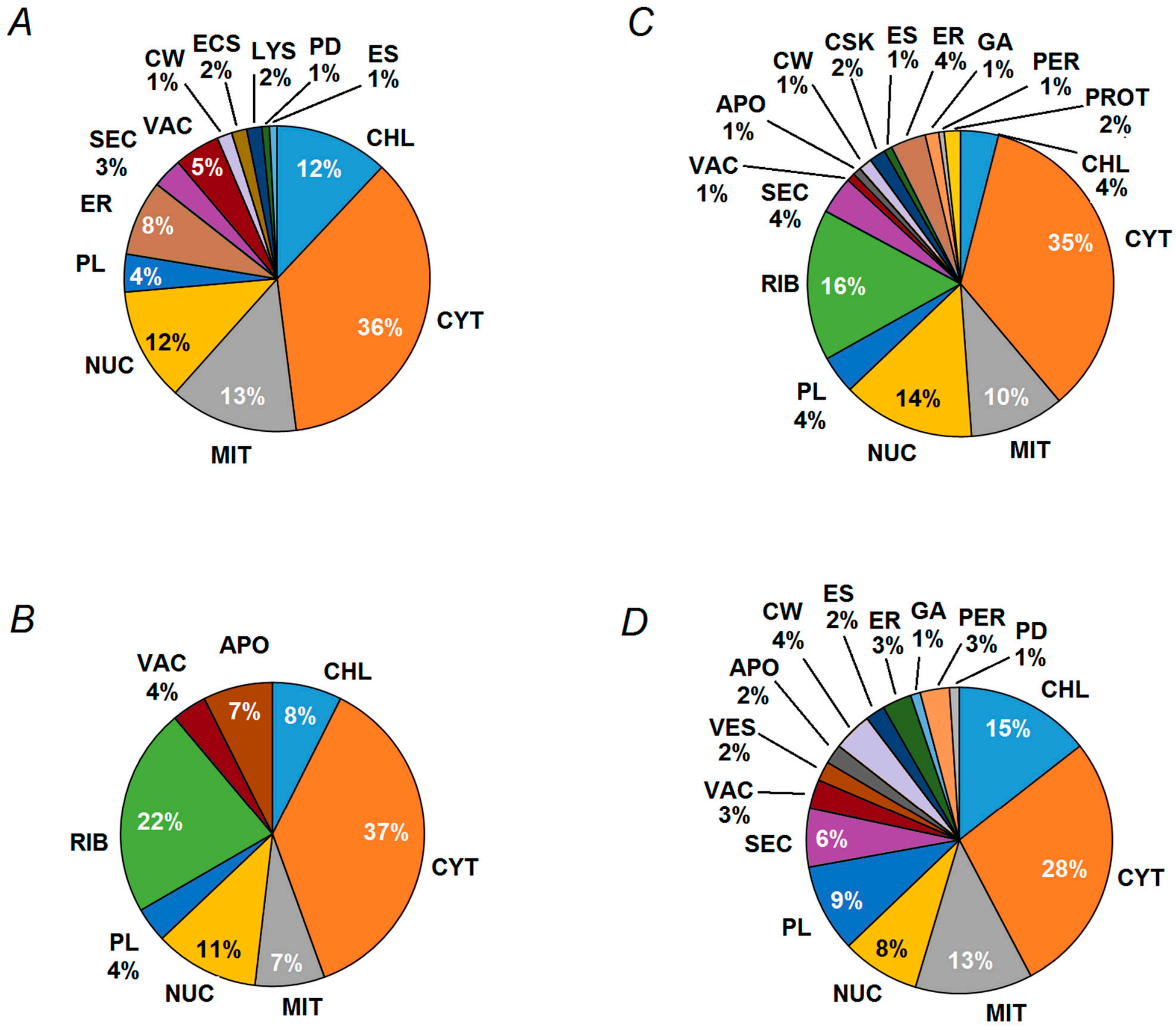

Figure S1-3 Prediction of intracellular localization for the differentially expressed proteins, annotated in the tryptic digests, obtained from *Raphanus sativus* plants, up-regulated (A,C) and down-regulated (B,D) on the 22th day after inoculation (d.a.i.) with cultural medium (A,B) and *Agrobacterium tumefaciens* (C,D) in comparison to the corresponding 10-day treatments. The prediction relied on the BUSCA tool and protein sequences from combined *Raphanus sativus* database (NCBI, Uniprot and 2015 *R. sativus* genome assembly), and was verified manually afterwards - the procedure relied on the accession-based search against free databases: UniprotKB (https://www.uniprot.org/), nextprot database (https://www.nextprot.org/), BRENDA Enzyme Database - BRENDA (brenda-enzymes.org).; APO – apoplast, CW – cell wall, CHL – chloroplast, CYT – cytoplasm, CSK – cytoskeleton, ES – endomembrane system, ESC - extracellular space, GA – Golgi apparatus, ER – endoplasmic reticulum, LD – lipid droplet, LYS – lysosome, MIT –

mitochondrion, NUC – nucleus, PD – plasmodesm, PER – peroxisome, PROT – proteasome, PL – plasma membrane, RIB – ribosome, SEC – secreted, VES - vesicle, VAC - vacuole

Figure S1-4 Tandem mass spectra of unic peptides representing the proteins listed in **Table 1**. The peptides were identified in a targeted SEQUEST search against a *R. sativum* database containing 37 selected sequences of peptides/proteins known to contribute in tumor growth and meristem function

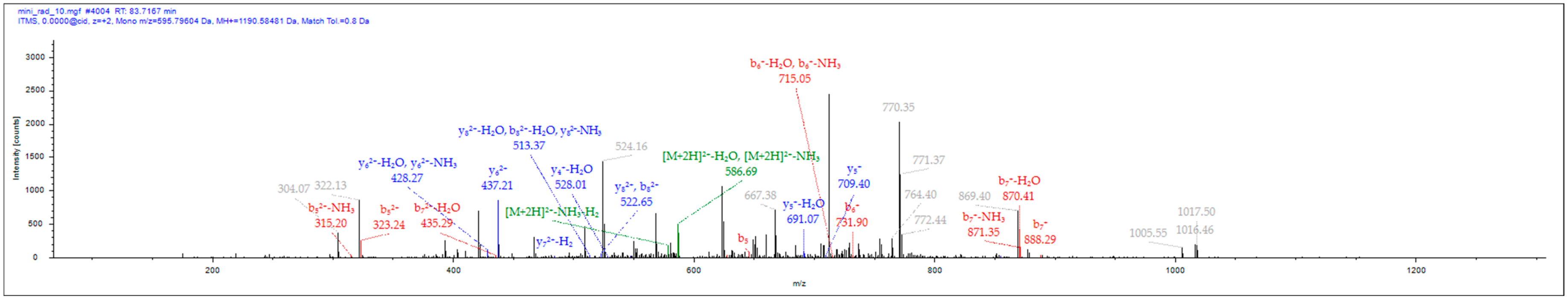

Figure S1-4.1 Tandem mass spectrum of the *m/z* 595.79 corresponding to the $[M+3H]^{3+}$ ion of the peptide MGNYYSRRK representing NP_680162.1 CLAVATA3/ESR-RELATED 22 (identified with 1 PSMs, XCorr = 0.03, PEP = 0.214)

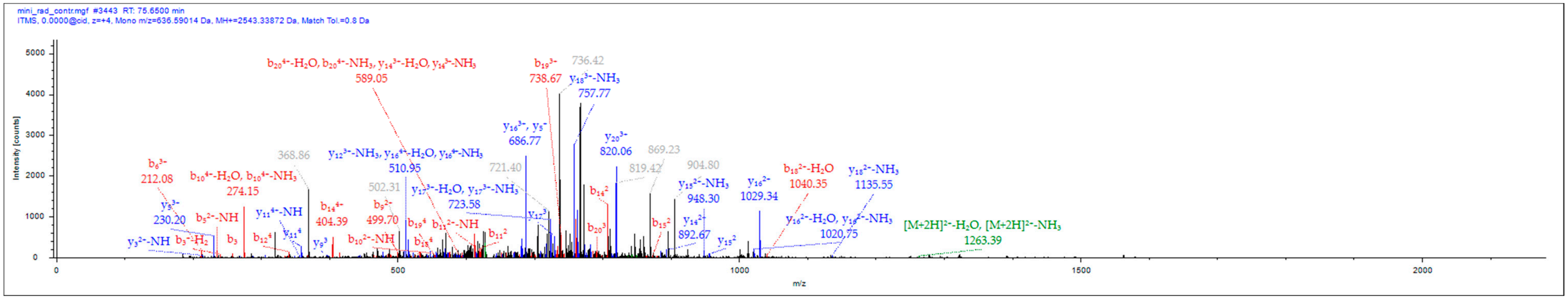

Figure S1-4.2 Tandem mass spectrum of the *m/z* 636.59 corresponding to the $[M+3H]^{3+}$ ion of the peptide SIGVQFKHTLEDQEMLNKNRR representing NP_001077799.1 CLAVATA3/ESR-RELATED 45 (identified with 4 PSMs, XCorr = 0.31, PEP = 0.153)

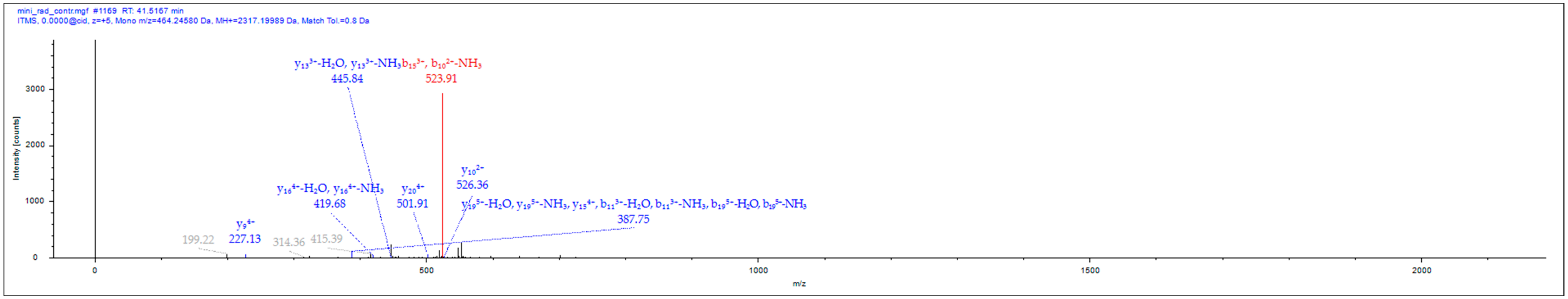

Figure S1-4.3 Tandem mass spectrum of the *m/z* 464.24 corresponding to the $[M+3H]^{3+}$ ion of the peptide YFGAGKFPPVDSFVGKGISESK representing NP_566783.1 CLAVATA3/ESR-RELATED 27 (identified with 4 PSMs, XCorr = 0.4, PEP = 0.0911)

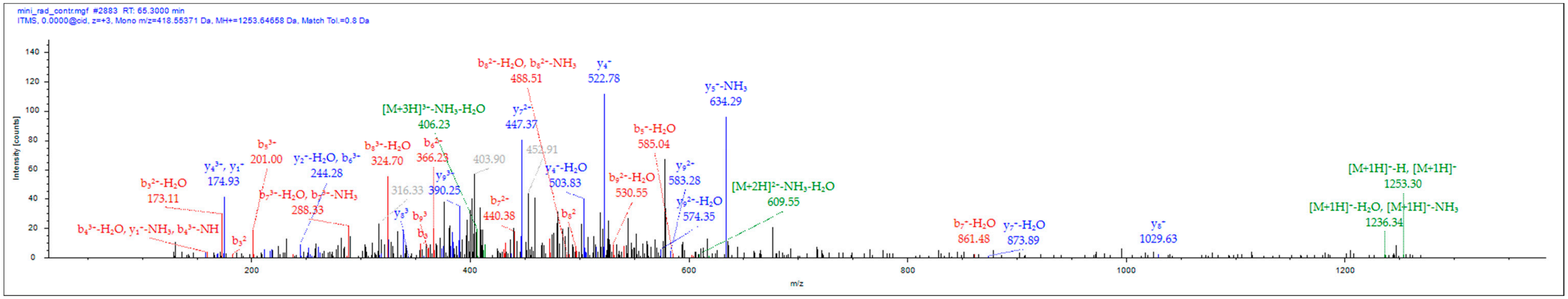

Figure S1-4.4 Tandem mass spectrum of the *m/z* 418.55 corresponding to the $[M+3H]^{3+}$ ion of the peptide SHHQLEFLSR representing NP_190576.1 CYCLIN D3;3 (identified with 1 PSMs, XCorr = 1.25, PEP = 0.0891)

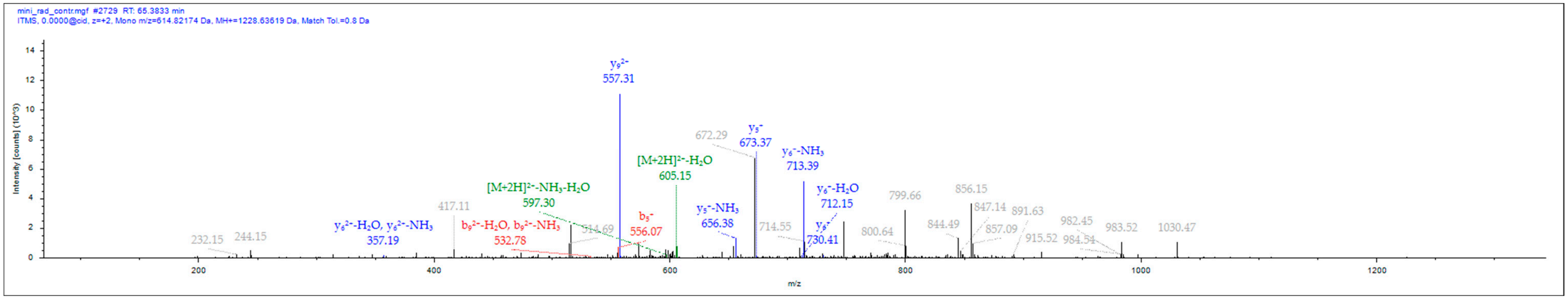

Figure S1-4.5.1 Tandem mass spectrum of the *m/z* 614.82 corresponding to the $[M+3H]^{3+}$ ion of the peptide NPMRGFHLEK representing NP_191622.1 GRAS family transcription factor (identified with 3 PSMs, XCorr = 0.2, PEP = 0.135)

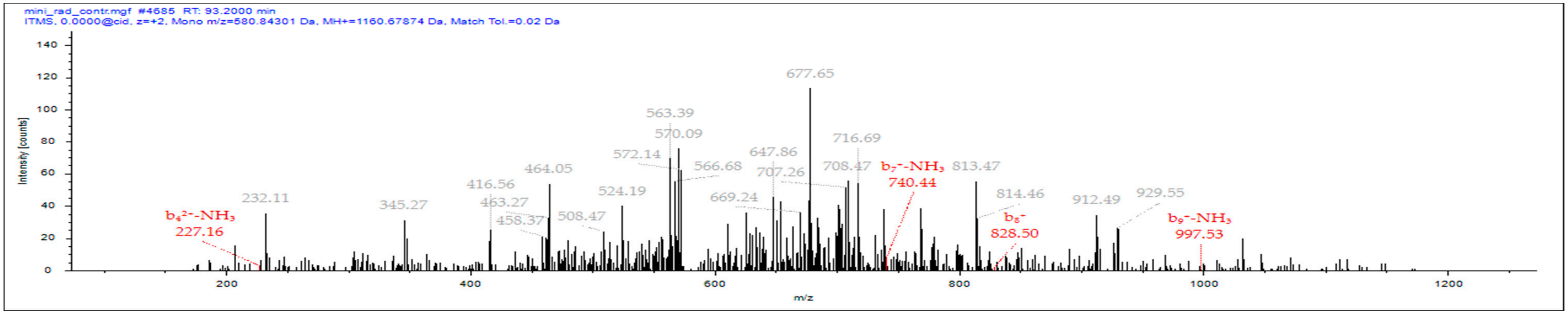

Figure S1-4.5.2 Tandem mass spectrum of the *m/z* 580.84 corresponding to the $[M+3H]^{3+}$ ion of the peptide KELVTVSAWK representing NP_191622.1 GRAS family transcription factor (identified with 1 PSMs, XCorr = 0.1, PEP =0.17)

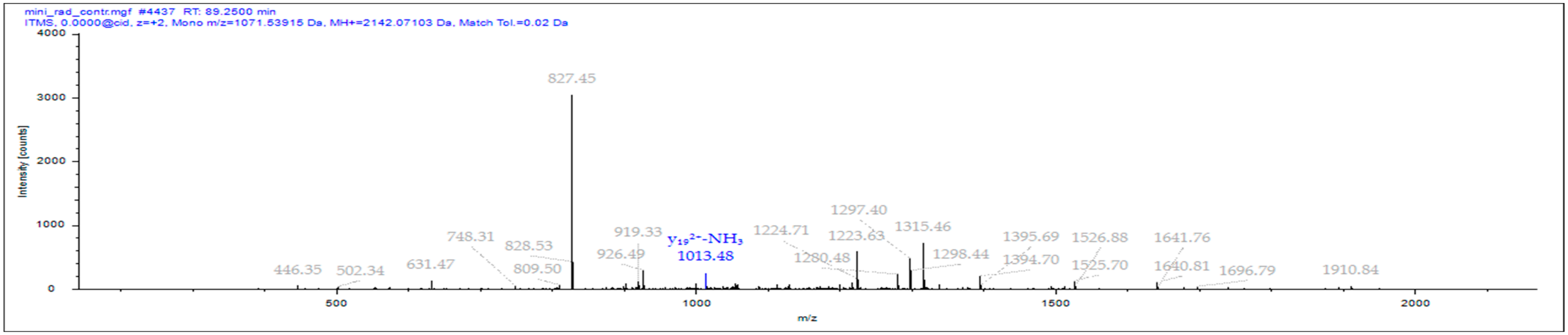

Figure S1-4.6 Tandem mass spectrum of the *m/z* 1071.54 corresponding to the $[M+3H]^{3+}$ ion of the peptide VVVFVDSEGWTEIAGSGSFR representing NP_195389.4 GRAS family transcription factor (identified with 6 PSMs, XCorr = 0.48, PEP = 0.123)

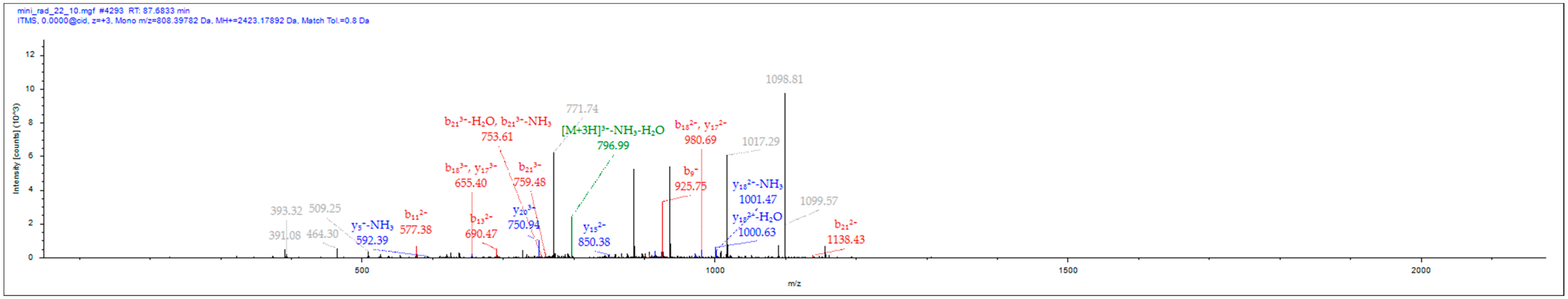

Figure S1-4.7 Tandem mass spectrum of the *m/z* 808.39 corresponding to the $[M+3H]^{3+}$ ion of the peptide AVSMGNMDSQVLLHELGFDLSK representing NP_850159.2 CLAVATA3/ESR-RELATED 5 (identified with 8 PSMs, XCorr = 0.68, PEP = 0.098)

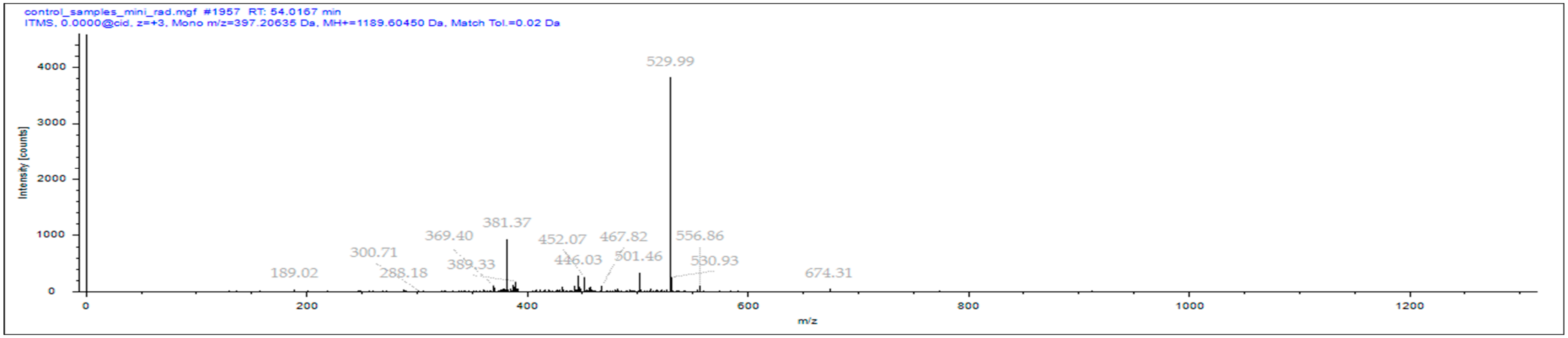

Figure S1-4.8.1 Tandem mass spectrum of the *m/z* 397.21 corresponding to the $[M+3H]^{3+}$ ion of the peptide SLYYKNHHK representing NP_001318877.1 CLAVATA3/ESR-RELATED 21 (identified with 1 PSMs, XCorr =0.06, PEP = 1.2e-1)

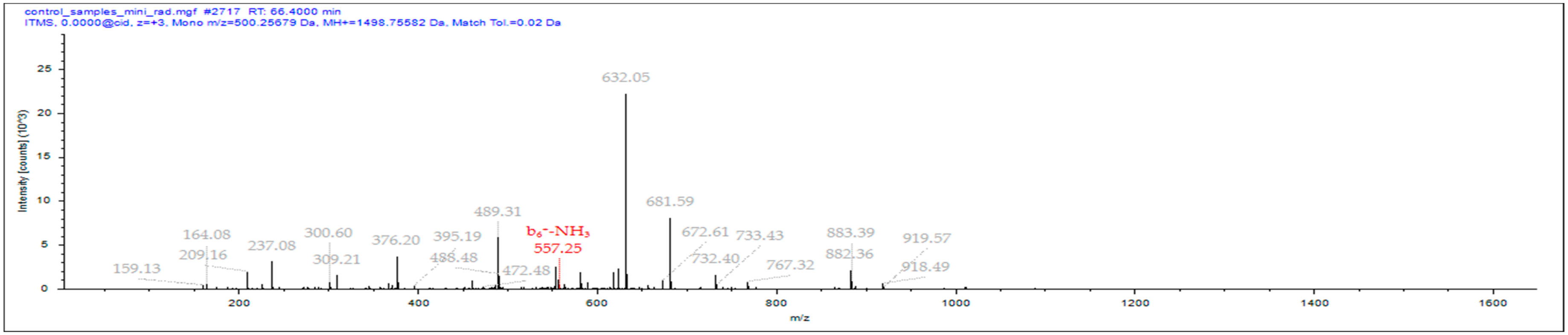

Figure S1-4.8.2 Tandem mass spectrum of the *m/z* 500.25 corresponding to the $[M+3H]^{3+}$ ion of the peptide SSSIQAGRFMTTGR representing NP_001318877.1 CLAVATA3/ESR-RELATED 21 (identified with 1 PSMs, XCorr = 0.3, PEP =3.2e-1)

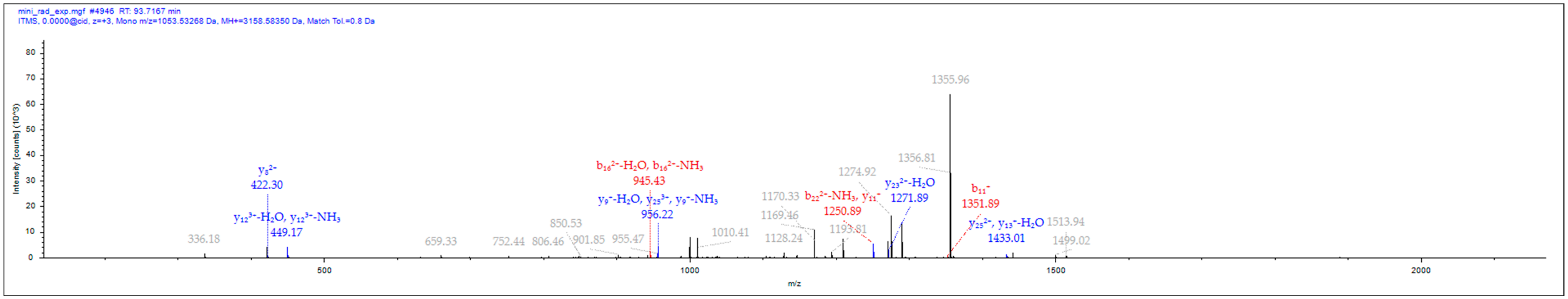

Figure S1-4.9 Tandem mass spectrum of the *m/z* 1053.53 corresponding to the $[M+3H]^{3+}$ ion of the peptide HRWTPTSTQLQILESIYDEGSGTPNRR representing NP_173493.2 WUSCHEL-related homeobox 14 (identified with 11 PSMs, XCorr =0.79, PEP = 0.334)

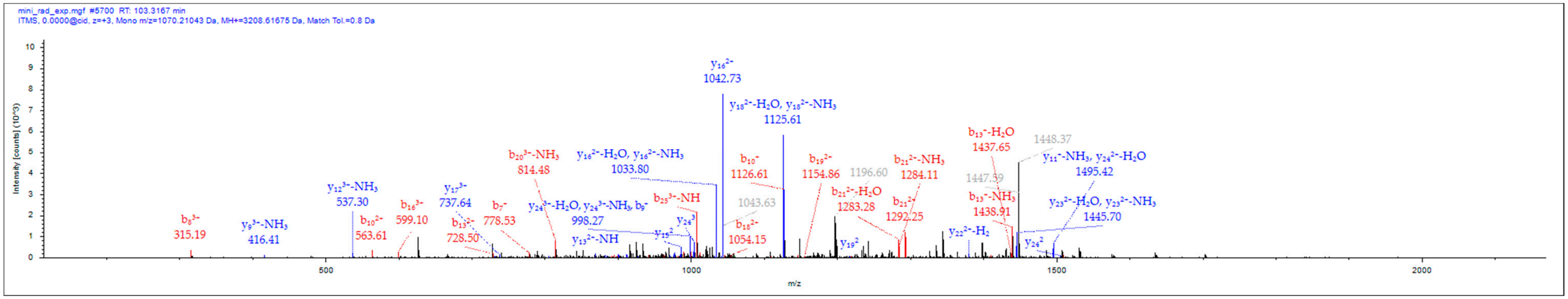

Figure S1-4.10 Tandem mass spectrum of the *m/z* 1070.21corresponding to the $[M+3H]^{3+}$ ion of the peptide ISTELSFYGKIESKNVFYWFQNHKAR representing NP_187735.2 WUSCHEL related homeobox 5 (identified with 1 PSMs, XCorr = 0.82, PEP = 0.287)

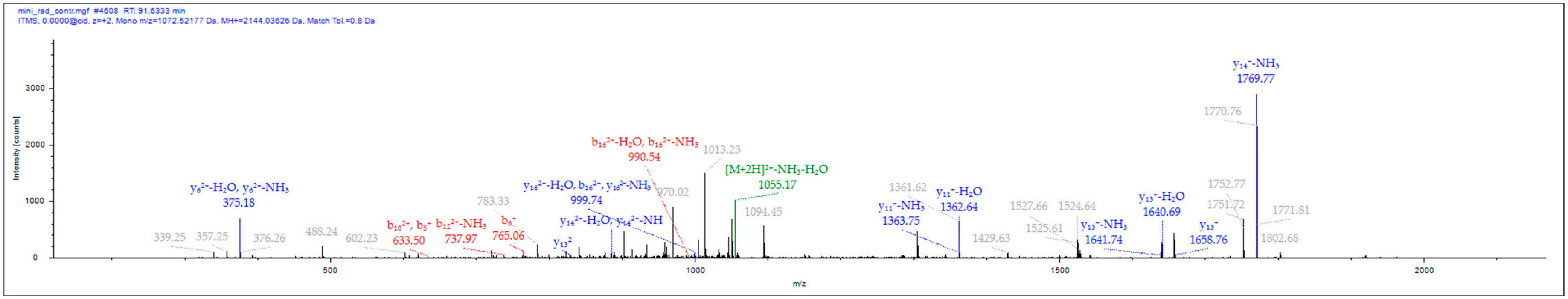

Figure S1-4.11 Tandem mass spectrum of the *m/z* 1072.52 corresponding to the $[M+3H]^{3+}$ ion of the peptide ELDQFMTHYVLLLCSFK representing NP_197904.1 homeobox protein knotted-1-like 3 (identified with 2 PSMs, XCorr = 0.46, PEP = 0.206)

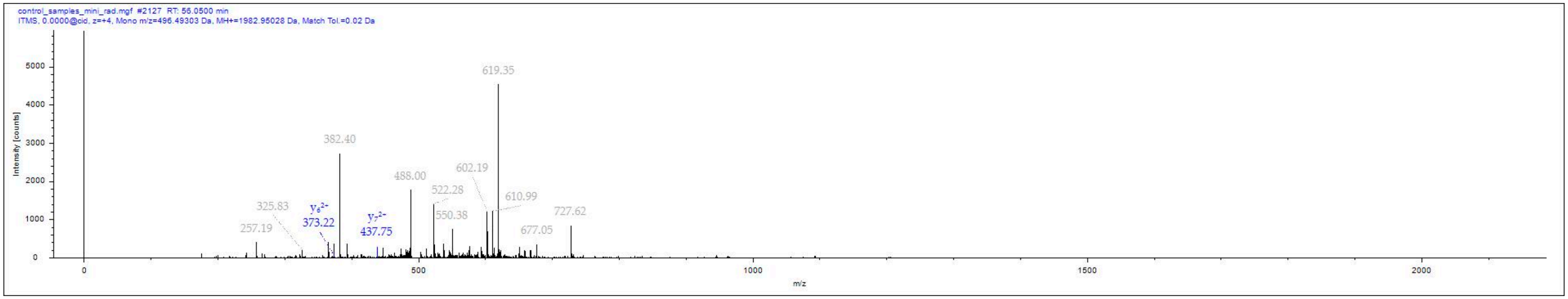

Figure S1-4.12 Tandem mass spectrum of the *m/z* 595.79 corresponding to the $[M+3H]^{3+}$ ion of the peptide SLLMNNGSYEEEEQVLK representing NP_683589.1 CLAVATA3/ESR-RELATED 19 (identified with 2 PSMs, XCorr = 0.47, PEP = 2.5e-1)

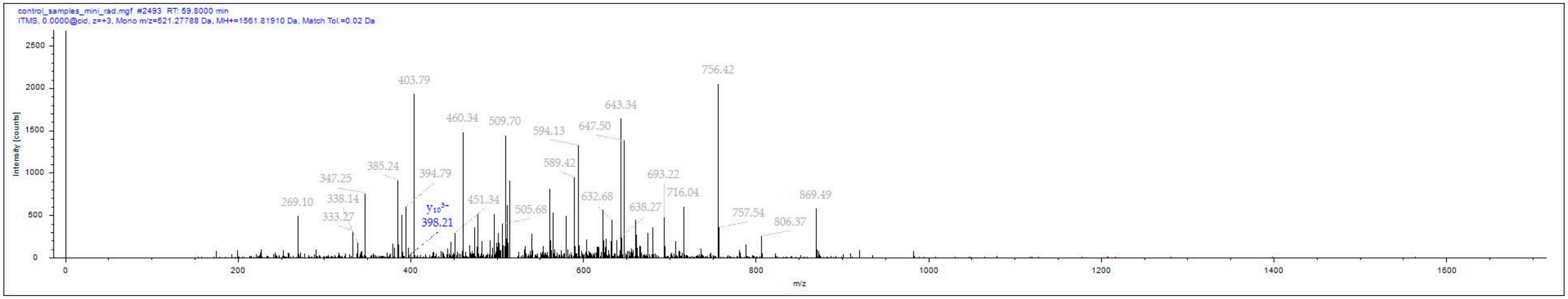

Figure S1-4.13 Tandem mass spectrum of the *m/z* 521.27 corresponding to the $[M+3H]^{3+}$ ion of the peptide RGRMMIEAEEVLK representing NP_001319370.1 CLAVATA3/ESR-RELATED 1 (identified with 3 PSMs, XCorr = 0.63, PEP = 2.2e-1)

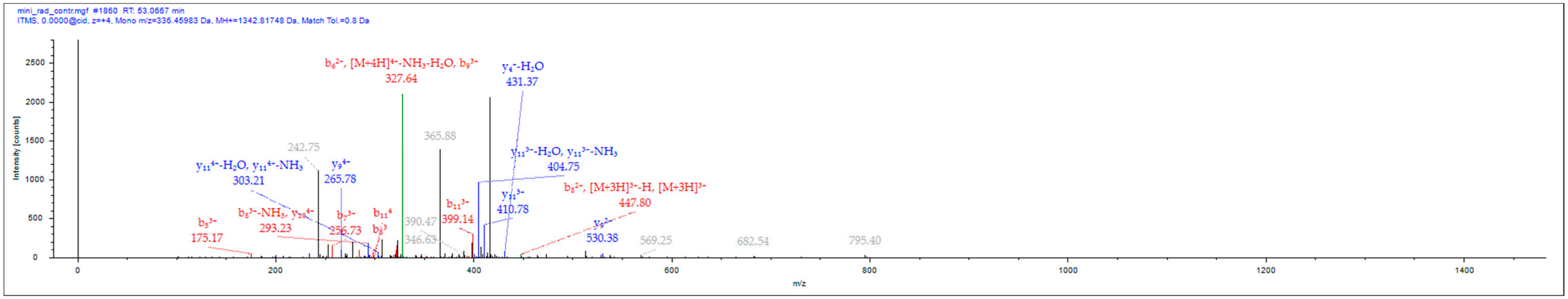

Figure S1-4.14 Tandem mass spectrum of the *m/z* 336.45 corresponding to the $[M+3H]^{3+}$ ion of the peptide IGLQIQIQSSKK representing NP_195142.1 CYCLIN D3;1 (identified with 1 PSMs, XCorr = 0.26, PEP = 0.186)

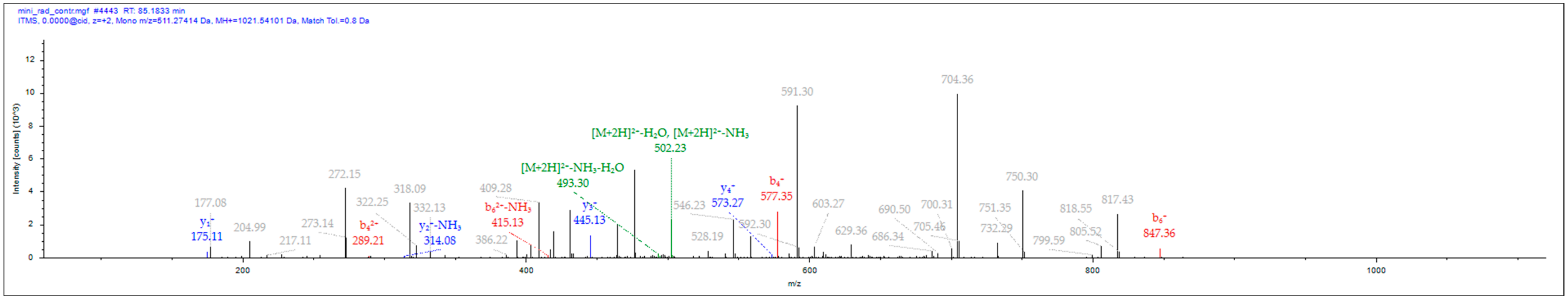

Figure S1-4.15 Tandem mass spectrum of the *m/z* 511.27 corresponding to the $[M+3H]^{3+}$ ion of the peptide VWFQNRR representing NP_195014.1 homeobox-leucine zipper protein ATHB-8 (identified with 7 PSMs, XCorr =0.94, PEP = 0.189)

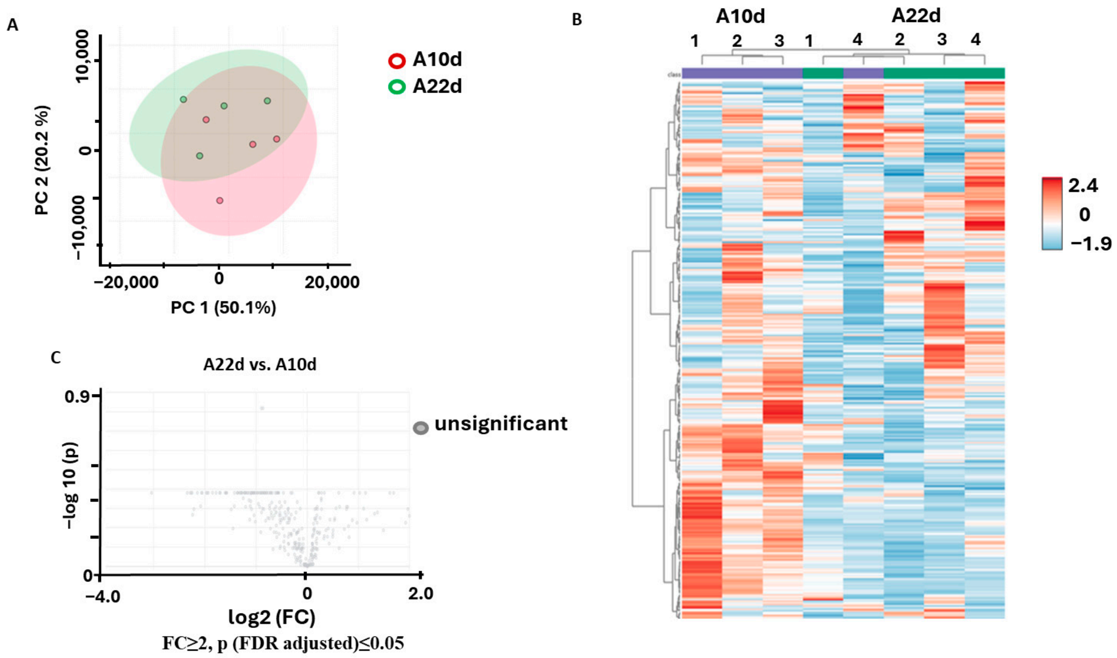

Figure S1-5. Comparison of the primary metabolite profiles detectable in aq. methanolic extracts prepared from the *R. sativus* calluses, cultivated on the agrobacteria cultural medium on the 10th d.a.i. - A10d and on the 22nd d.a.i. – A22d. Statistical analysis relied on the principal component analysis (PCA) with a score plot representation (A), hierarchical clustering analysis with a heatmap representation (B) and volcano plot with a graphical representation of differentially abundant analytes - (C) with Benjamini–Hochberg false discovery rate (FDR) correction at $p \leq 0.05$ and fold change (FC) ≥ 2. The metabolites marked with grey dots showed no statistically significant differences.

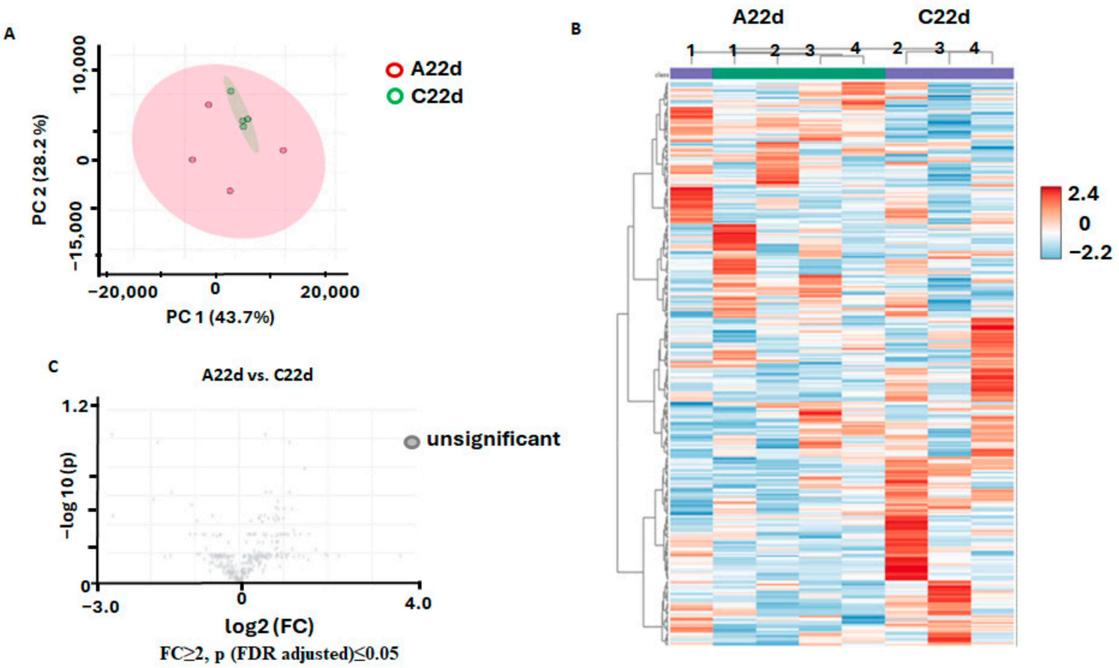

Figure S1-6. Comparison of the primary metabolite profiles detectable in aq. methanolic extracts prepared from the *R. sativus* calluses. The calluses were harvested on the 22nd day after inoculation with a suspension of the *A. tumefaciens* culture (A22d) or corresponding mock-treated controls (C22d - exposure to the bacteria-free cultural medium). Statistical analysis relied on the principal component analysis (PCA) with a score plot representation (A), hierarchical clustering analysis with a heatmap representation (B) and volcano plot with a graphical representation of differentially abundant analytes - (C) with Benjamini–Hochberg false discovery rate (FDR) correction at $p \leq 0.05$ and fold change (FC) ≥ 2. The metabolites marked with grey dots showed no statistically significant differences
